# Structural Characterization and Visible-Light Photocatalytic Performance of AgBr-Modified Zr-Containing Titanium Silicalite-1 (TS-1) Composites

**DOI:** 10.3390/molecules31213921

**Published:** 2026-10-30

**Authors:** Hristina Lazarova, Elena Tacheva, Borislav Barbov, Rusi Rusew, Stela Atanasova-Vladimirova, Boris Shivachev

**Affiliations:** 1 Institute of Mineralogy and Crystallography “Acad. Ivan Kostov”, Bulgarian Academy of Sciences, 1113 Sofia, Bulgaria; tacheva_e@imc.bas.bg (E.T.); barbov@imc.bas.bg (B.B.); r.rusev93@imc.bas.bg (R.R.); 2 PERIMED-2, BG16RFPR002-1.014-0007, Central District, Vasil Aprilov Blvd. 15A, 4002 Plovdiv, Bulgaria; 3 National Centre of Excellence Mechatronics and Clean Technologies, 8 bul. Kliment Ohridski, 1756 Sofia, Bulgaria; 4 Institute of Physical Chemistry, Bulgarian Academy of Sciences, 1113 Sofia, Bulgaria

**Keywords:** wastewater treatment, titanium silicate 1 (TS-1), AgBr modified, Zr incorporation, photocatalytic degradation, methyl orange

## Abstract

AgBr-modified Zr-containing titanium silicalite-1 (TS-1) composites were prepared and evaluated for methyl orange (MO) removal under white-light irradiation. AgBr modification markedly enhanced the visible-light response of the Zr-containing TS-1 materials, resulting in MO removal efficiencies of up to 98.6% after 50 min of irradiation. Structural and textural characterization confirmed preservation of the MFI framework after modification, while diffuse-reflectance measurements showed that the visible-light absorption of the composites is predominantly governed by the AgBr component. The composites retained substantial activity over five consecutive cycles, and scavenger experiments indicated a possible contribution of hydroxyl radicals to MO removal. These results demonstrate that AgBr modification is an effective strategy for enhancing the visible-light activity of Zr-containing TS-1 materials.

## 1. Introduction

The continuous discharge of dye-containing industrial wastewaters from textile, paper, leather, and printing industries represents a significant environmental challenge because many synthetic dyes are chemically stable, poorly biodegradable, and potentially toxic to aquatic ecosystems and human health [1,2]. Among these pollutants, methyl orange (MO), a widely used azo dye, is frequently employed as a model contaminant due to its high stability, complex aromatic structure, and resistance to conventional biological treatment processes. Consequently, the development of efficient and environmentally friendly technologies for MO degradation has attracted considerable research interest. Advanced oxidation processes, particularly semiconductor photocatalysis, have emerged as promising approaches because they enable the complete mineralization of organic pollutants under mild operating conditions without generating secondary contaminants [3,4,5].

Titanium silicalite-1 (TS-1) is one of the most important titanium-containing zeolites owing to its MFI framework, high thermal and chemical stability, large specific surface area, and the presence of isolated tetrahedrally coordinated Ti species. These characteristics make TS-1 an excellent catalyst for selective oxidation reactions and an attractive candidate for photocatalytic applications. However, the relatively wide band gap of TS-1 limits its absorption mainly to the ultraviolet region, resulting in poor utilization of visible light and rapid recombination of photogenerated electron–hole pairs. Considerable efforts have therefore been devoted to improving the photocatalytic efficiency of TS-1 through framework modification, heteroatom incorporation, and the functionalization of photoactive materials such as silver-based compounds [6,7,8].

Among various modification strategies, zirconium incorporation into the TS-1 framework has attracted increasing attention because Zr species can modify the local electronic environment of Ti active sites, improve framework stability, alter the textural properties, and facilitate charge separation during photocatalysis. Moderate Zr incorporation has been reported to enhance photocatalytic activity, whereas excessive Zr loading may decrease performance due to framework distortion, partial blockage of active sites, and increased charge-carrier recombination [9,10,11].

Another effective strategy for improving visible-light photocatalysis is coupling TS-1 with narrow-band-gap semiconductors. Silver bromide (AgBr) is considered one of the most attractive visible-light-responsive photocatalysts because of its suitable band-gap energy (~2.5–2.7 eV) [12], excellent visible-light absorption, and efficient generation of reactive oxygen species. Furthermore, the modification with AgBr enhances charge separation and suppresses the recombination of photogenerated electron–hole pairs, thereby improving the photocatalytic performance of the material [5,13]. In addition, partial photoreduction of AgBr can generate metallic Ag nanoparticles, whose localized surface plasmon resonance further enhances visible-light absorption and photocatalytic activity [14,15]. As a result, AgBr-based composites have demonstrated excellent performance in the degradation of various organic dyes under visible-light irradiation [16,17].

Although numerous AgBr-based photocatalysts have been reported, studies combining AgBr modified with zirconium-incorporation TS-1 are not available. Most previous investigations have focused either on Zr-incorporation TS-1 without a visible-light sensitizer or on AgBr coupled with conventional semiconductor photocatalysts such as TiO_2_, Bi-based oxides, g-C_3_N_4_-based, or silver halide systems [18,19,20,21]. Therefore, the synergistic effects arising from the combination of framework-modified TS-1 and AgBr have not yet been systematically investigated [11,22,23,24,25].

The present study investigates the effect of AgBr modification on the structural, textural, optical, and methyl orange removal properties of previously synthesized Zr-containing TS-1 materials [9]. Two composites, TS-1/0.5Zr/AgBr and TS-1/1Zr/AgBr, were prepared and characterized using complementary analytical techniques. Particular attention was given to the contribution of adsorption and the additional MO removal observed under white-light irradiation, as well as to the influence of zirconium content on the performance of the resulting materials.

## 2. Results and Discussion

The Zr-containing TS-1 materials (TS-1/0.5Zr and TS-1/1Zr), previously synthesized and characterized in our earlier study [9], were used as starting materials in the present work. These materials were subsequently modified by deposition of AgBr to enhance their visible-light response. The resulting TS-1/0.5Zr/AgBr and TS-1/1Zr/AgBr composites were evaluated for methyl orange (MO) removal under white-light irradiation. The present study therefore focuses primarily on the effect of AgBr modification on the structural, optical, and photocatalytic properties of the previously characterized Zr-containing TS-1 materials.

### 2.1. Physicochemical Characterization of TS-1/xZr/AgBr Form

The elemental composition of the synthesized materials, determined by EDXRF analysis, is summarized in Table 1. The parent TS-1 contained Si (91.53 wt%) and Ti (8.47 wt%), consistent with the expected composition of titanium silicalite. In the modified composites, Zr, Ag, and Br were clearly detected in addition to Si and Ti. The Zr content increased from 0.32 wt% in TS-1/0.5Zr/AgBr to 0.67 wt% in TS-1/1Zr/AgBr, consistent with the higher nominal Zr content of the starting materials. The Ag content was 11.02 and 13.08 wt% for TS-1/0.5Zr/AgBr and TS-1/1Zr/AgBr, respectively, while the corresponding Br contents were 14.44 and 14.05 wt%. Trace amounts of K (0.10–0.12 wt%) were also detected and can be attributed to residual species originating from the KBr precursor used during AgBr deposition. Thus, EDXRF analysis confirms the presence of Zr, Ag, and Br in the modified TS-1 materials, consistent with the preparation of the TS-1/Zr/AgBr composites. Because the elemental concentrations were obtained by standardless EDXRF analysis of loose powders, the reported values should be regarded as semi-quantitative; consequently, deviations in the apparent Ag/Br ratio were not interpreted stoichiometrically. The formation of crystalline AgBr in the composites is independently confirmed by the PXRD results discussed below.

**Figure 1:**
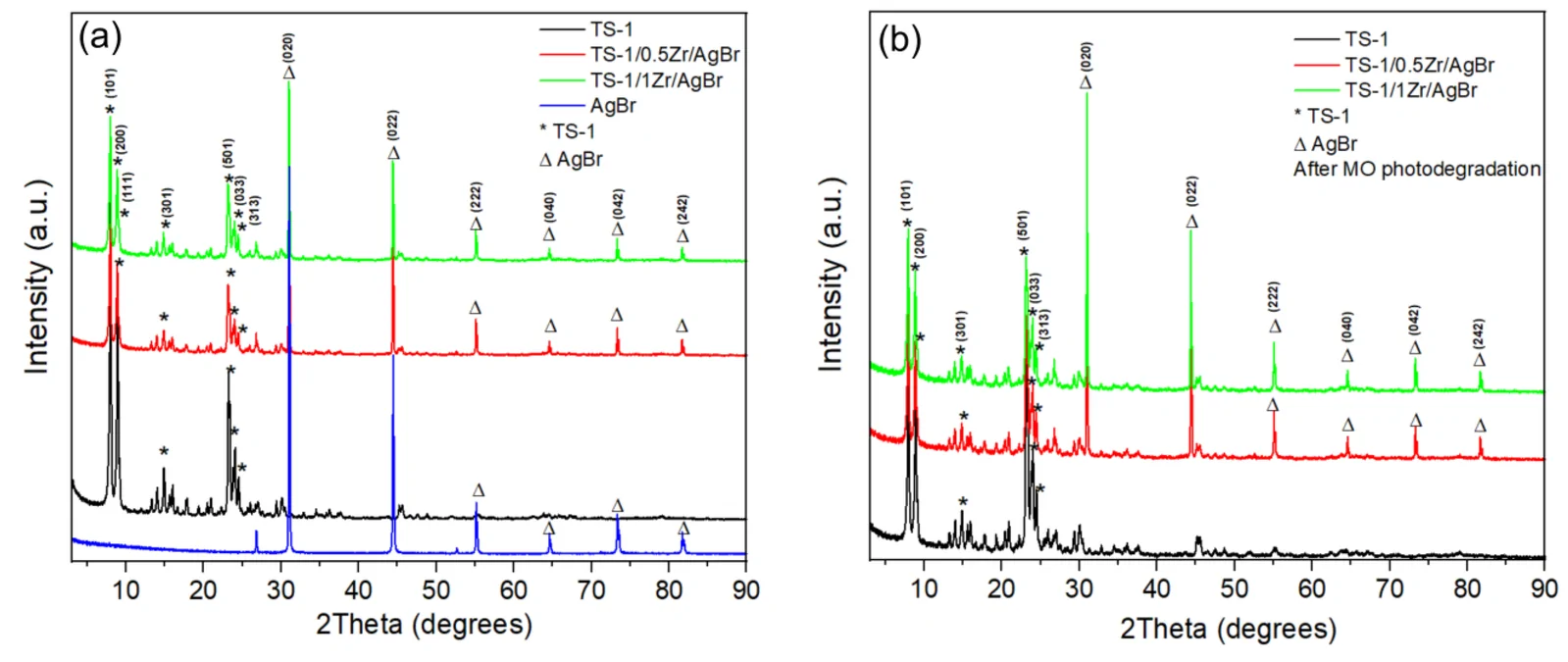
PXRD patterns of TS-1, TS-1/0.5Zr/AgBr, and TS-1/1Zr/AgBr (**a**) before and (**b**) after photocatalytic use under white-light irradiation. The characteristic reflections of the MFI framework and crystalline AgBr are indicated by asterisks (*) and triangles (△), respectively. The structural characterization of the parent TS-1 support was previously reported in Ref. [9].

The crystalline structure and phase composition of the investigated materials were examined by powder X-ray diffraction (PXRD), and the corresponding patterns are presented in Figure 1. The diffraction pattern of the parent TS-1 support exhibits the characteristic reflections of the MFI framework at approximately 2θ = 7.9°, 8.8°, 23.1°, 23.9°, and 24.4° (PDF No. 01-089-8099) [26], consistent with our previously reported results [9]. These reflections remain clearly detectable in both TS-1/0.5Zr/AgBr and TS-1/1Zr/AgBr, indicating that the MFI crystalline framework is preserved following AgBr modification. In addition to the characteristic MFI reflections, both composites exhibit diffraction peaks at approximately 2θ = 30.9°, 44.3°, 55.0°, 64.4°, 73.1°, and 81.2°, corresponding to the cubic AgBr phase (PDF No. 96-150-9152) [27]. The presence of these reflections confirms the formation of crystalline AgBr in the modified materials. The relatively higher intensity of the AgBr reflections in TS-1/1Zr/AgBr is consistent with its higher Ag content determined by XRF analysis. No distinct reflections attributable to crystalline ZrO_2_ (PDF No. 00-037-1484) [28] were identified, which may be associated with the low zirconium content (0.32 and 0.67 wt.% for TS-1/0.5Zr/AgBr and TS-1/1Zr/AgBr, respectively), the high dispersion of Zr-containing species, or their possible amorphous nature. To evaluate the structural stability of the AgBr-containing composites and assess possible photocorrosion during photocatalysis, PXRD patterns were also recorded after 50 min of white-light irradiation during MO removal (Figure 1b). The characteristic reflections of both the MFI framework and crystalline AgBr remain detectable after the photocatalytic experiments, indicating retention of the principal crystalline phases. Furthermore, no clearly distinguishable new diffraction reflections attributable to crystalline metallic Ag were observed within the detection limits of PXRD. These observations suggest that no substantial bulk crystalline phase transformation occurred during the investigated irradiation period. Nevertheless, minor photoreduction of AgBr to metallic Ag or surface-localized photocorrosion cannot be excluded solely on the basis of PXRD analysis. Therefore, the results support the short-term preservation of the crystalline structure under the investigated conditions but do not establish complete resistance to photocorrosion or long-term operational stability.

**Figure 2:**
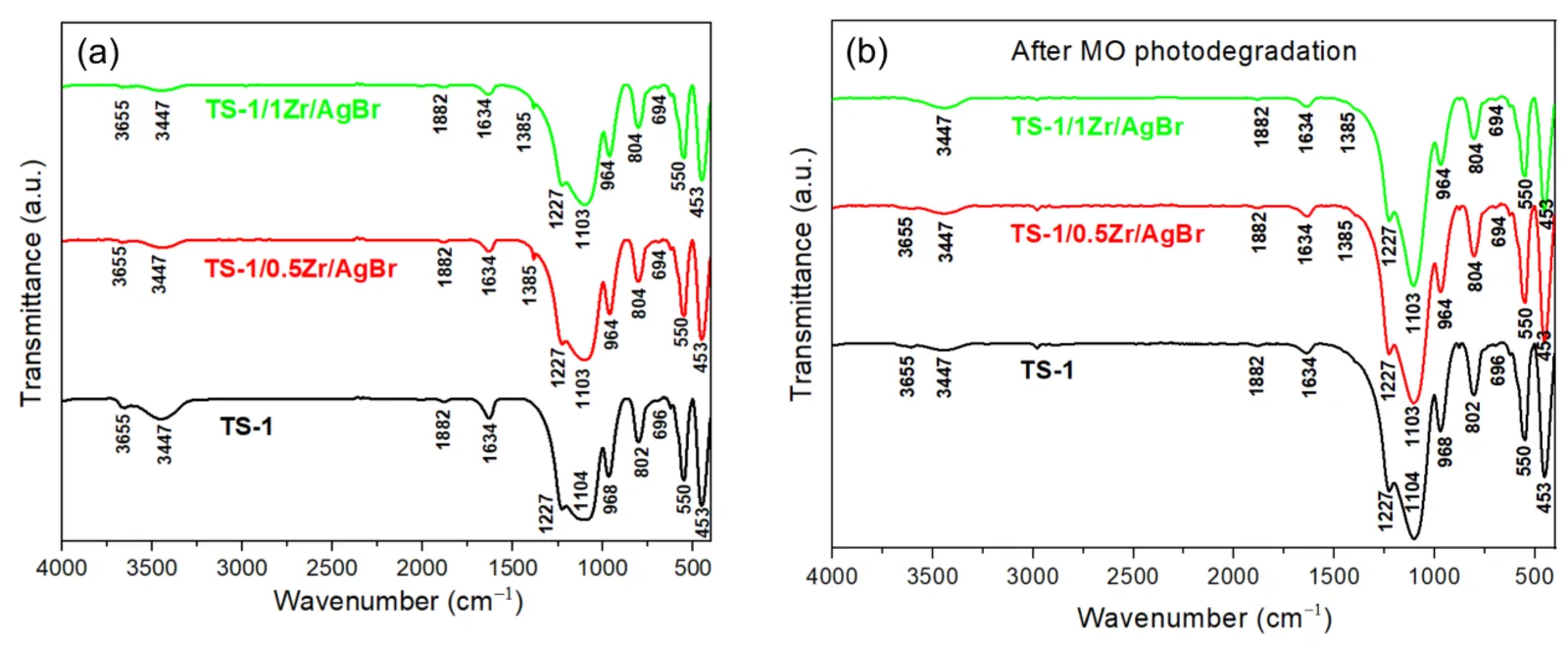
FTIR spectra of TS-1, TS-1/0.5Zr/AgBr, and TS-1/1Zr/AgBr (**a**) before and (**b**) after photocatalytic use for methyl orange removal under white-light irradiation. The characteristic vibrational bands associated with the titanium silicalite framework remain detectable after photocatalytic treatment. The FTIR characterization of the parent TS-1 support was previously reported in Ref. [9].

The FTIR spectra of TS-1, TS-1/0.5Zr/AgBr, and TS-1/1Zr/AgBr are presented in Figure 2. All samples exhibit the characteristic vibrational bands of the MFI framework. The broad bands at 3655–3445 cm^−1^ and the band at approximately 1634 cm^−1^ are attributed to surface hydroxyl groups and adsorbed water molecules. The intense bands at around 1227 and 1103–1104 cm^−1^ correspond to the asymmetric stretching vibrations of Si–O–Si and Si–O–Ti linkages within the zeolite framework. The band near 964 cm^−1^ is associated with Si–O–Ti-related vibrations [29,30], while those at 802–804 and 451–453 cm^−1^ are assigned to symmetric stretching and bending vibrations of Si–O–Si linkages, respectively [29]. The bands near 550 and 1230 cm^−1^ were also associated with the MFI structure. These assignments are further consistent with the FTIR analysis of MFI-type zeolite [31].

The weak absorption feature at 1882–1884 cm^−1^, observed in all three samples, including the unmodified TS-1 support, is assigned to an overtone of framework Si–O vibrations. This assignment is supported by Chen et al. [32], who attributed a band at approximately 1882 cm^−1^ in highly siliceous HZSM-5 to a Si–O overtone of the zeolite framework. Therefore, the presence of this band cannot be specifically associated with AgBr modification. In contrast, the band at approximately 1358 cm^−1^ appears only in the AgBr-modified composites. This feature may be associated with residual species introduced during the AgBr deposition process, potentially including nitrate-containing species originating from the AgNO_3_ precursor. Since the available FTIR data do not permit an unambiguous assignment, this interpretation is presented as tentative. Comparison of the FTIR spectra before and after MO photodegradation (Figure 2b) reveals that the principal vibrational features associated with the MFI framework remain detectable following photocatalytic treatment. No major changes in the positions of the characteristic framework bands are apparent, suggesting that the local bonding environment of the TS-1 framework is largely preserved under the investigated conditions. These observations are consistent with the PXRD results (Figure 1), which indicate retention of the principal crystalline phases after irradiation. Nevertheless, FTIR spectroscopy alone cannot exclude subtle surface modifications or partial chemical transformations of the AgBr component during photocatalysis.

**Figure 3:**
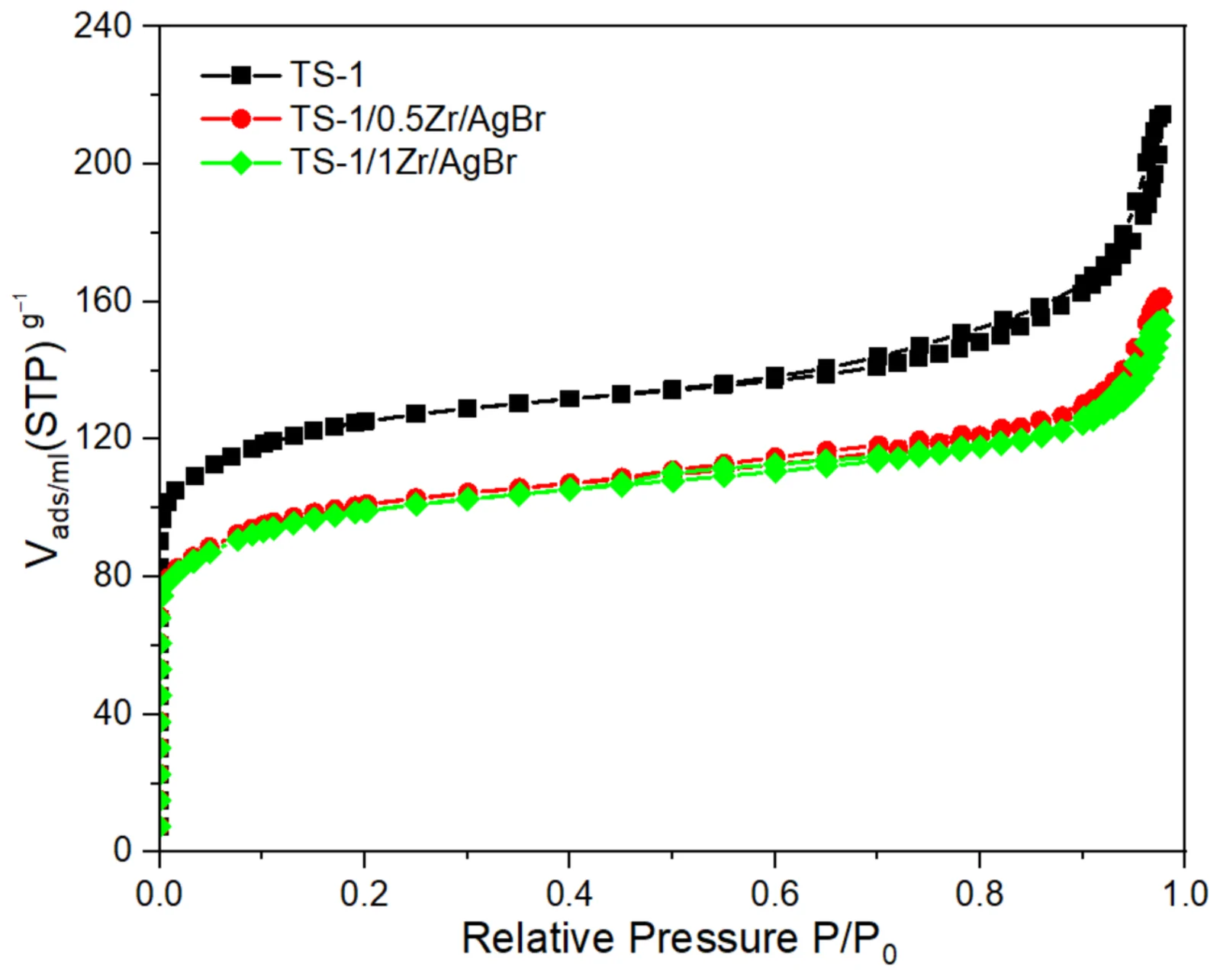
N_2_ adsorption–desorption isotherms showing the textural properties of TS-1 and AgBr-modified Zr-incorporation TS-1 composites. The textural properties of the parent TS-1 support were previously reported in Ref. [9].

The N_2_ adsorption–desorption isotherms and the corresponding textural parameters of the synthesized materials are presented in Figure 3 and Table 2, respectively. Consistent with the FTIR and PXRD results, the textural analysis indicates that AgBr modification of the Zr-containing TS-1 materials did not substantially alter the fundamental porous architecture of TS-1, although changes in accessible surface area and microporosity were observed. The parent TS-1 exhibited a high BET specific surface area (S_BET_) of **414.52** m^2^ g^−1^, a micropore surface area (S_micro_) of 270.82 m^2^ g^−1^, and a micropore volume (V_micro_) of 0.13 cm^3^ g^−1^, which are characteristic of highly crystalline MFI-type zeolites. Following Zr incorporation and AgBr modification, a decrease in both the specific surface area and microporosity was observed. The S_BET_ values decreased to **330.15 and 324.33** m^2^ g^−1^ for TS-1/0.5Zr/AgBr and TS-1/1Zr/AgBr, respectively, while the micropore surface area decreased to **204.65 and 199.26 m^2^ g^−1^.** Similarly, the micropore volume decreased from 0.13 cm^3^ g^−1^ for TS-1 to 0.10 cm^3^ g^−1^ for both modified samples. The reduction in surface area and micropore volume can be attributed to the modification of AgBr particles and the incorporation of zirconium species, which partially occupy the zeolite pores and cover part of the external surface. Nevertheless, the total pore volume remained relatively high (0.22–0.23 cm^3^ g^−1^), and the BJH-derived average pore diameter (apparent mesopore diameter) showed only a slight change from 2.8 to 2.7 nm. These results indicate that the porous architecture of TS-1 was largely preserved after modification. The observed decrease in specific surface area and micropore volume is therefore attributed primarily to partial pore blocking and surface coverage by AgBr particles rather than to framework degradation, in agreement with the FTIR and PXRD results confirming the preservation of the MFI structure.

**Figure 4:**
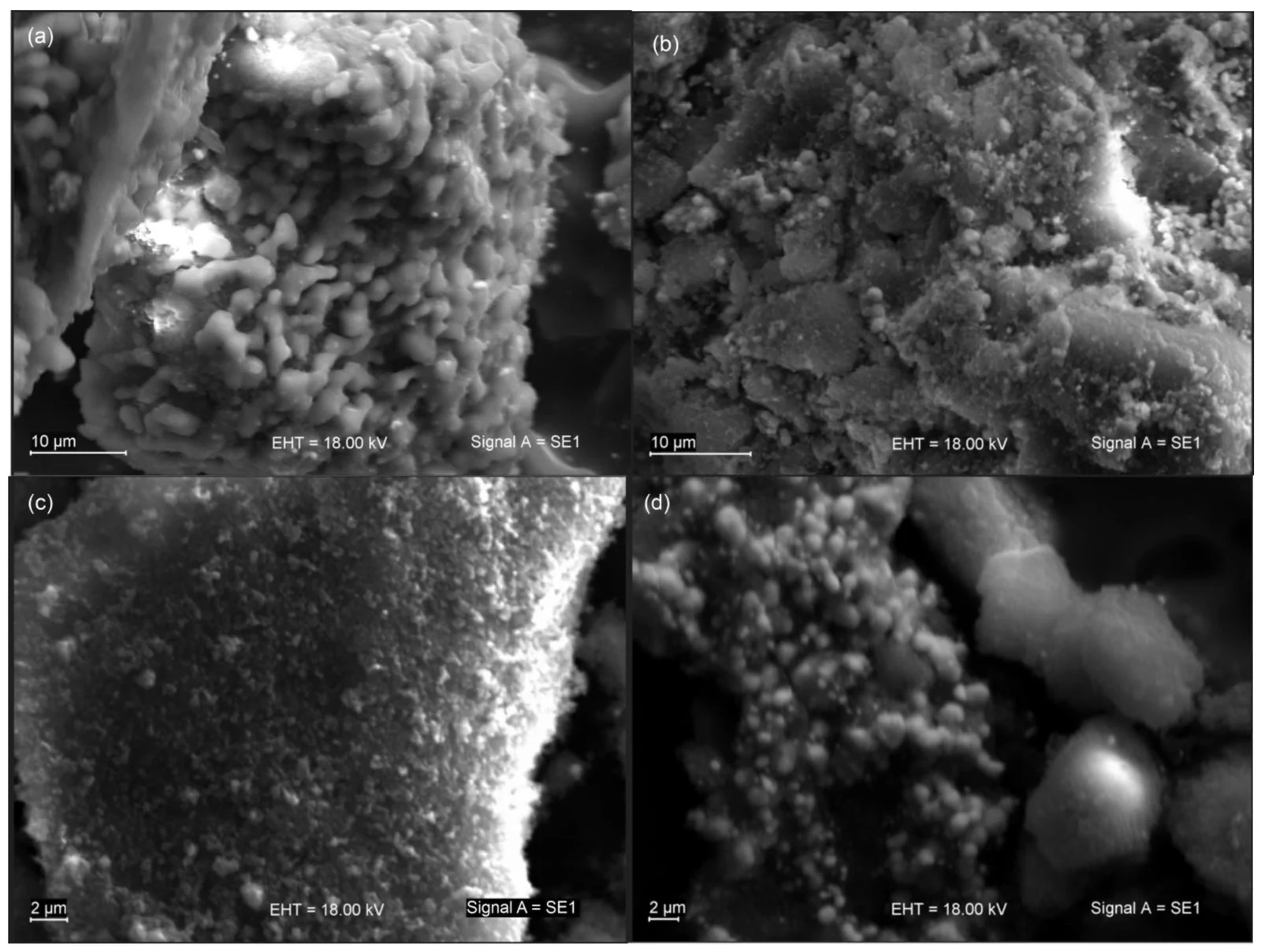
SEM images of TS-1/0.5Zr/AgBr at different magnifications: (**a**) scale bar, 10 μm; (**b**) scale bar, 10 μm; (**c**) scale bar, 2 μm; (**d**) scale bar, 2 μm, illustrating the mor-phology, aggregation, and surface texture of the composite particles.

SEM analysis (Figure 4) was performed to investigate the morphology of the TS-1/0.5Zr/AgBr composite. At lower magnifications (Figure 4a,b), the material exhibits irregularly shaped particles forming agglomerates with heterogeneous surface textures. At higher magnifications (Figure 4c,d), smaller morphological features are visible on the surfaces of the larger aggregates. However, the SEM images alone do not permit an unambiguous identification of these features as AgBr nanoparticles or an assessment of their spatial distribution. The presence of crystalline AgBr is independently supported by the PXRD results, while the EDXRF analysis confirms the presence of Ag and Br in the composite.

**Figure 5:**
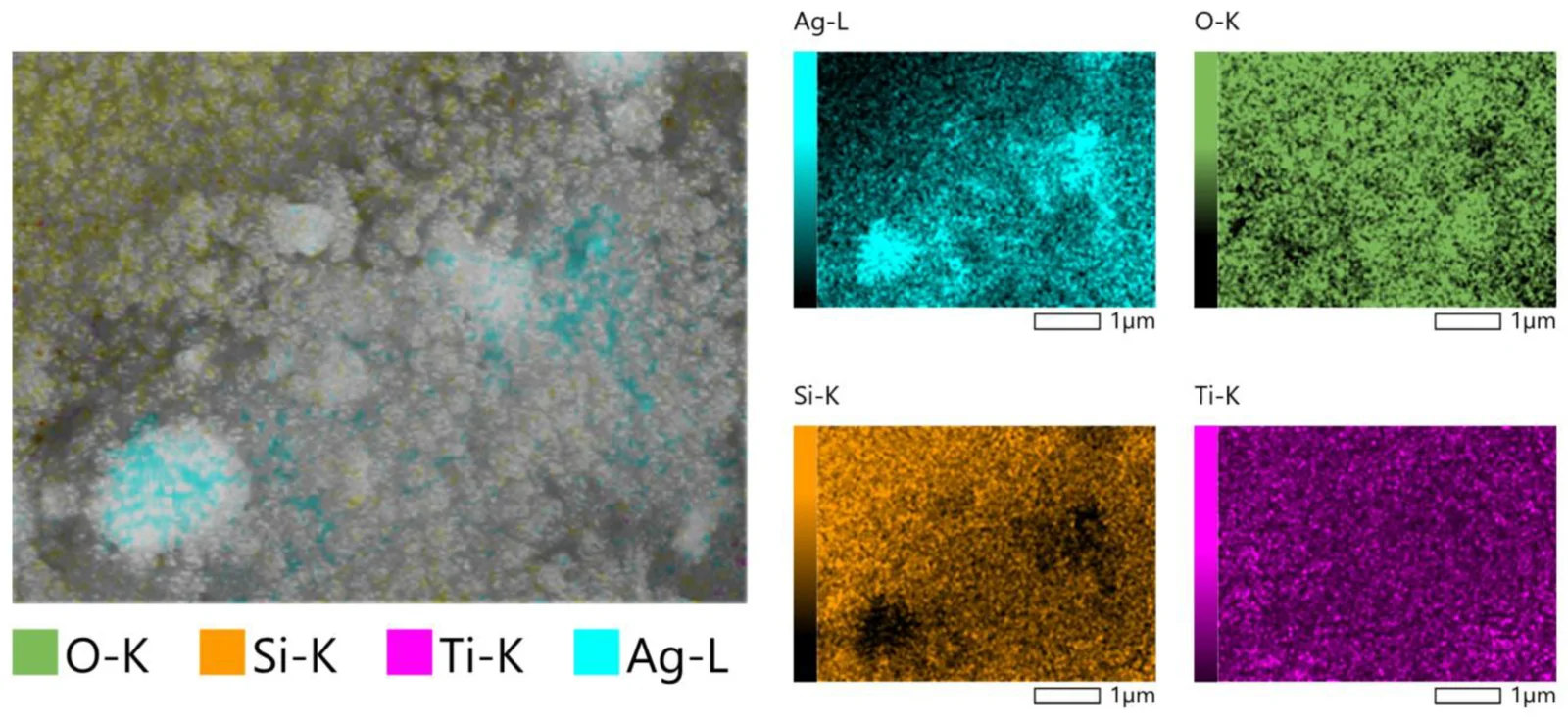
SEM–EDS elemental mapping of the TS-1/0.5Zr/AgBr composite. The SEM image with superimposed elemental distributions is shown on the left, while the individual elemental maps of Ag (Ag-L), O (O-K), Si (Si-K), and Ti (Ti-K) are presented on the right. The maps illustrate the distribution of the principal elements within the analyzed region, including localized Ag-enriched domains.

To further investigate the elemental distribution within the TS-1/0.5Zr/AgBr composite, SEM coupled with energy-dispersive X-ray spectroscopy (SEM–EDS) was employed (Figure 5). The elemental maps confirm the presence of Si, Ti, O, and Ag within the analyzed region (Figure S1). Silicon, titanium, and oxygen signals are distributed throughout the investigated area, consistent with the presence of the titanium silicate framework. In contrast, the Ag signal exhibits a heterogeneous spatial distribution, with localized regions of increased intensity, suggesting the presence of Ag-enriched domains. These observations provide additional information on the distribution of silver-containing species within the composite. However, the elemental maps alone do not establish the chemical state of Ag or unambiguously identify the Ag-rich regions as AgBr. The presence of crystalline AgBr is independently supported by the PXRD results.

**Figure 6:**
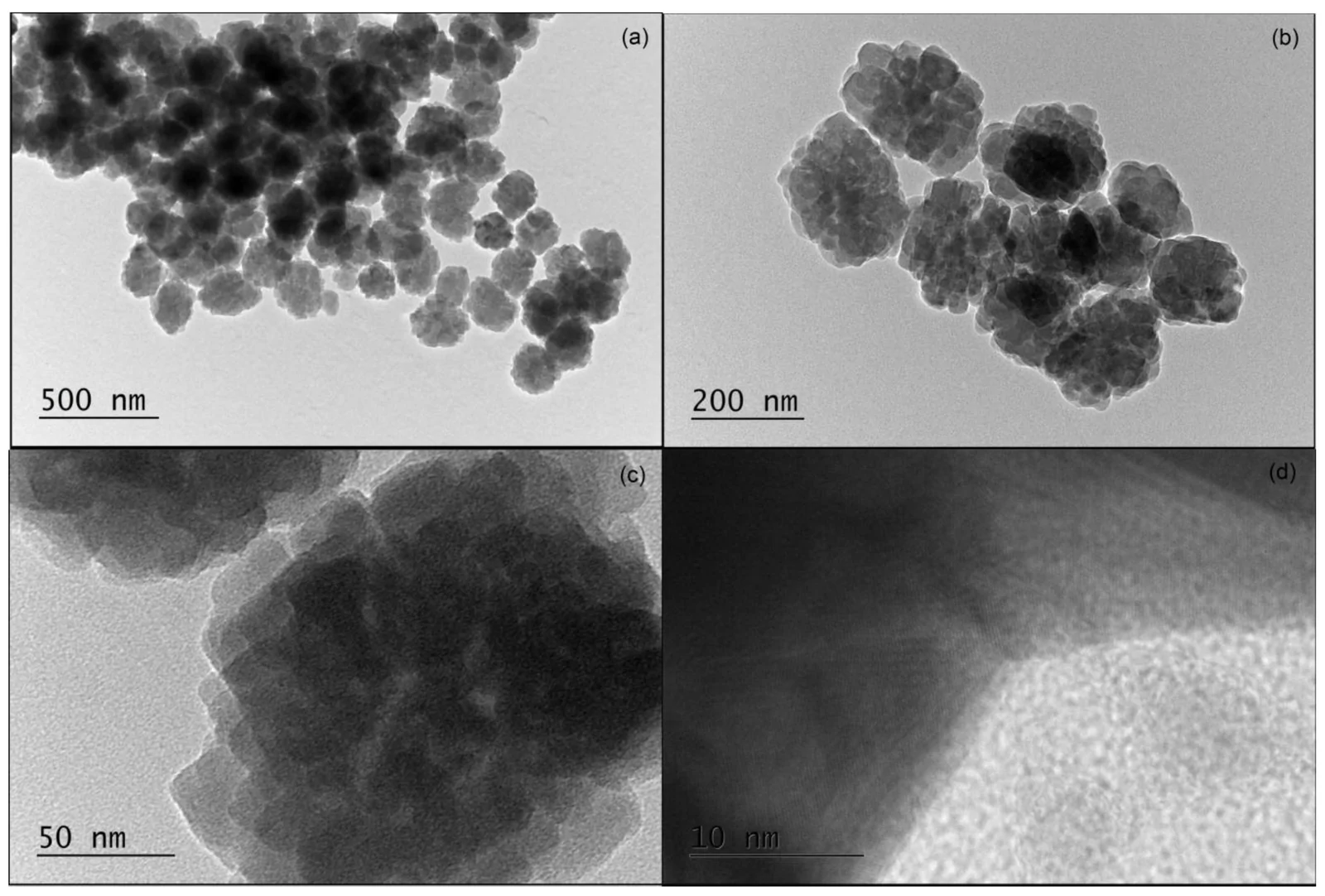
TEM and HRTEM images of the TS-1/0.5Zr/AgBr composite at different magnifications: (**a**,**b**) particle morphology and aggregation; (**c**) overlapping particles within an aggregate; and (**d**) high-resolution image showing localized lattice contrast.

Transmission electron microscopy (TEM) was employed to investigate the morphology and microstructure of the TS-1/0.5Zr/AgBr composite (Figure 6). The TEM images reveal aggregates composed of particles with different sizes and morphologies. At higher magnification, localized lattice fringes are observed, indicating crystalline ordering within selected regions of the material. However, the available TEM images do not allow the observed crystalline domains to be assigned unambiguously to TS-1 or AgBr. Furthermore, the spatial distribution of AgBr and the nature of its interface with the TS-1 support cannot be established from these observations alone. Additional analysis of selected HRTEM regions and their corresponding FFT patterns is presented in Figure 6.

**Figure 7:**
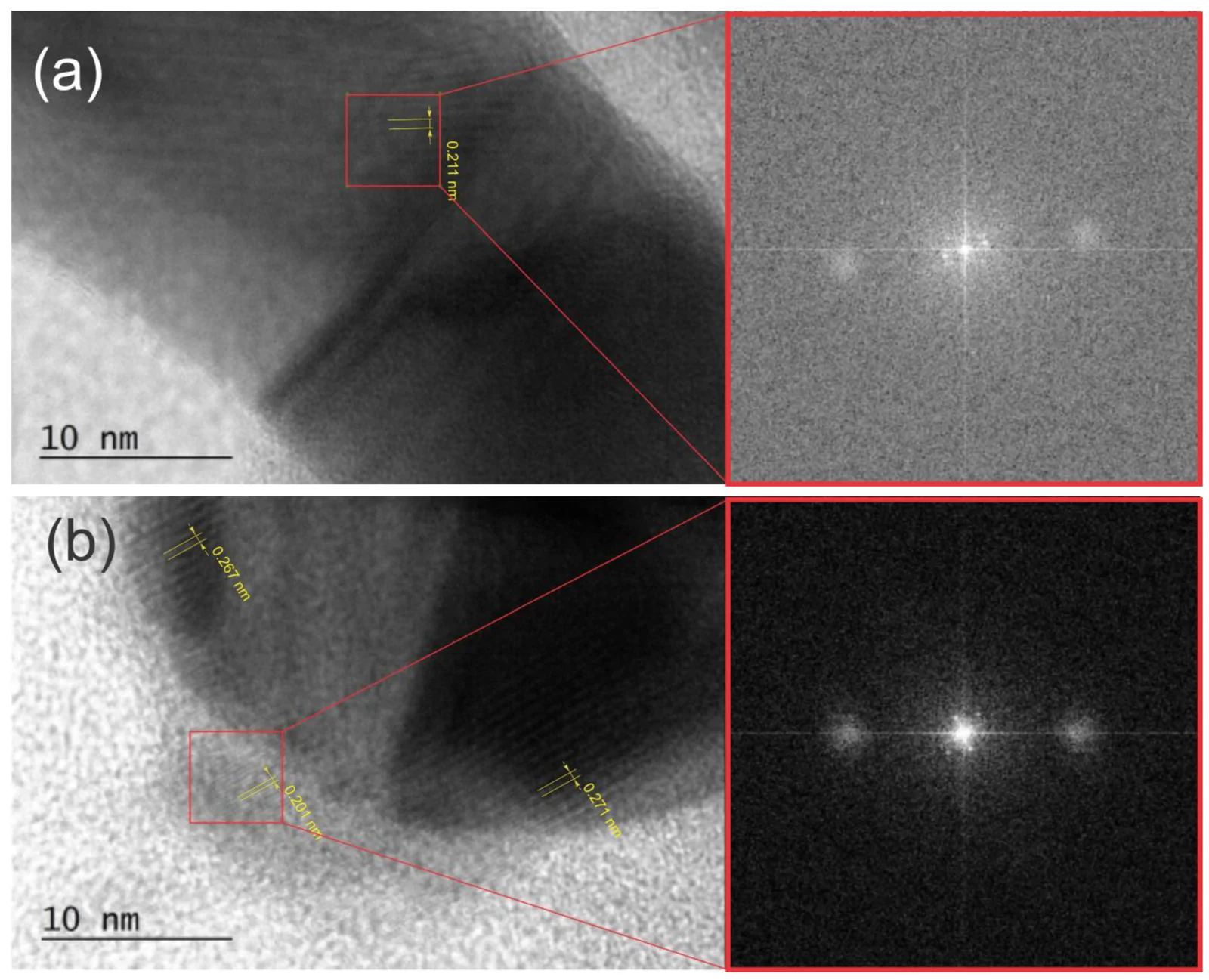
HRTEM micrographs of the AgBr-modified Zr-containing TS-1 composite showing localized lattice fringes with estimated interplanar spacings of (**a**) 0.211 nm and (**b**) 0.267, 0.201, and 0.271 nm. The corresponding FFT patterns of the selected regions are shown on the right. The observed lattice spacings may tentatively be attributed to TS-1 and/or AgBr crystalline domains, without an unambiguous phase assignment.

The HRTEM images (Figure 7) reveal localized lattice fringes, indicating the presence of crystalline domains within the investigated composites. The estimated interplanar spacings are approximately 0.211 nm (Figure 7a) and 0.267, 0.201, and 0.271 nm (Figure 7b). The corresponding fast Fourier transform (FFT) patterns exhibit localized intensity maxima consistent with periodic lattice contrast. The estimated interplanar spacings may be consistent with reflections from the TS-1 framework and/or crystalline AgBr. However, the limited precision of the measurements and the absence of sufficient crystallographic information prevent an unambiguous phase assignment. Therefore, these observations support the presence of local crystalline ordering but do not independently establish the identity of the crystalline domains or the formation of a well-defined AgBr/TS-1 interface.

### 2.2. Kinetic Studies

**Figure 8:**
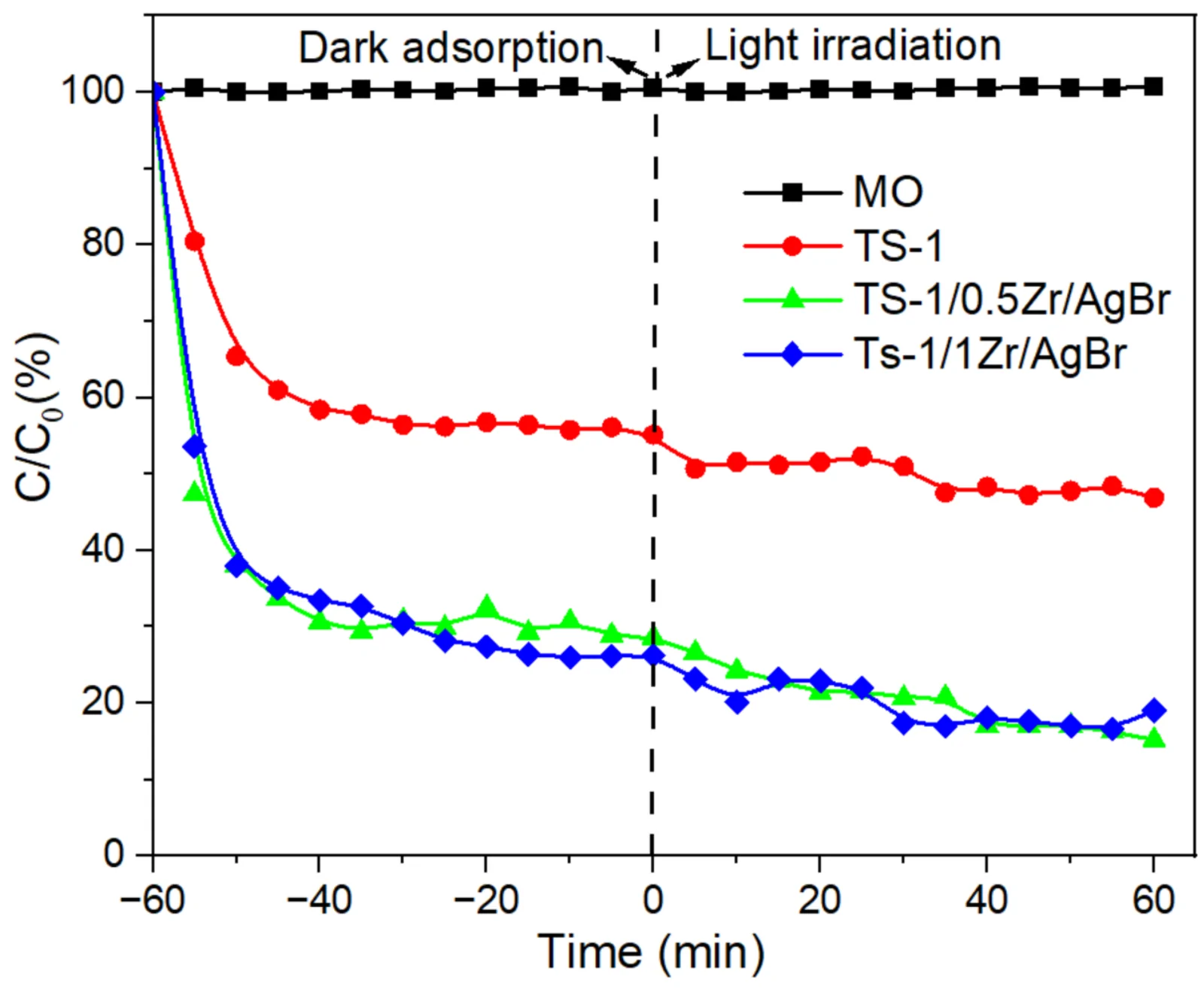
Time-dependent methyl orange removal in the presence of TS-1, TS-1/0.5Zr/AgBr, and TS-1/1Zr/AgBr during dark adsorption (−60–0 min) and subsequent white-light irradiation (0–60 min). The dashed vertical line indicates the onset of irradiation after the dark equilibration period. The relative MO concentration (C/C_initial_) was estimated from UV–Vis absorbance measurements at 464 nm.

To distinguish the contribution of dark adsorption from the subsequent removal of methyl orange (MO) under white-light irradiation, the catalyst suspensions were maintained under dark conditions for 60 min prior to illumination. The evolution of MO absorbance at 464 nm during dark adsorption and after the subsequent 60-min irradiation period is summarized in Table S1. The corresponding MO removal efficiencies during the dark adsorption and irradiation stages are presented in Table S2. During the initial 60-min dark adsorption period, MO removal efficiencies of 44.9%, 71.4%, and 73.7% were obtained for TS-1, TS-1/0.5Zr/AgBr, and TS-1/1Zr/AgBr, respectively. These results indicate substantially higher MO adsorption by the AgBr-modified composites compared with the parent TS-1 support. Following dark adsorption, the suspensions were exposed to white-light irradiation for an additional 60 min. During this stage, the MO concentration decreased by a further 15.0%, 46.9%, and 27.6% for TS-1, TS-1/0.5Zr/AgBr, and TS-1/1Zr/AgBr, respectively, relative to the MO concentration remaining at the end of the dark adsorption period (Table S2). Thus, TS-1/0.5Zr/AgBr exhibited the highest additional MO removal during irradiation, despite TS-1/1Zr/AgBr showing slightly greater adsorption under dark conditions. The evolution of the normalized MO concentration during the sequential dark adsorption and white-light irradiation experiments is presented in Figure 8. During the initial 60-min dark adsorption period (−60 to 0 min), the MO concentration decreased markedly in the presence of both AgBr-modified composites, whereas a less pronounced decrease was observed for the parent TS-1. After switching on the light (t = 0 min), a further decrease in MO concentration was observed, particularly for TS-1/0.5Zr/AgBr. In contrast, the MO control experiment performed without a catalyst showed negligible concentration changes throughout the experiment. These observations are consistent with the removal efficiencies summarized in Table S2.

**Figure 9:**
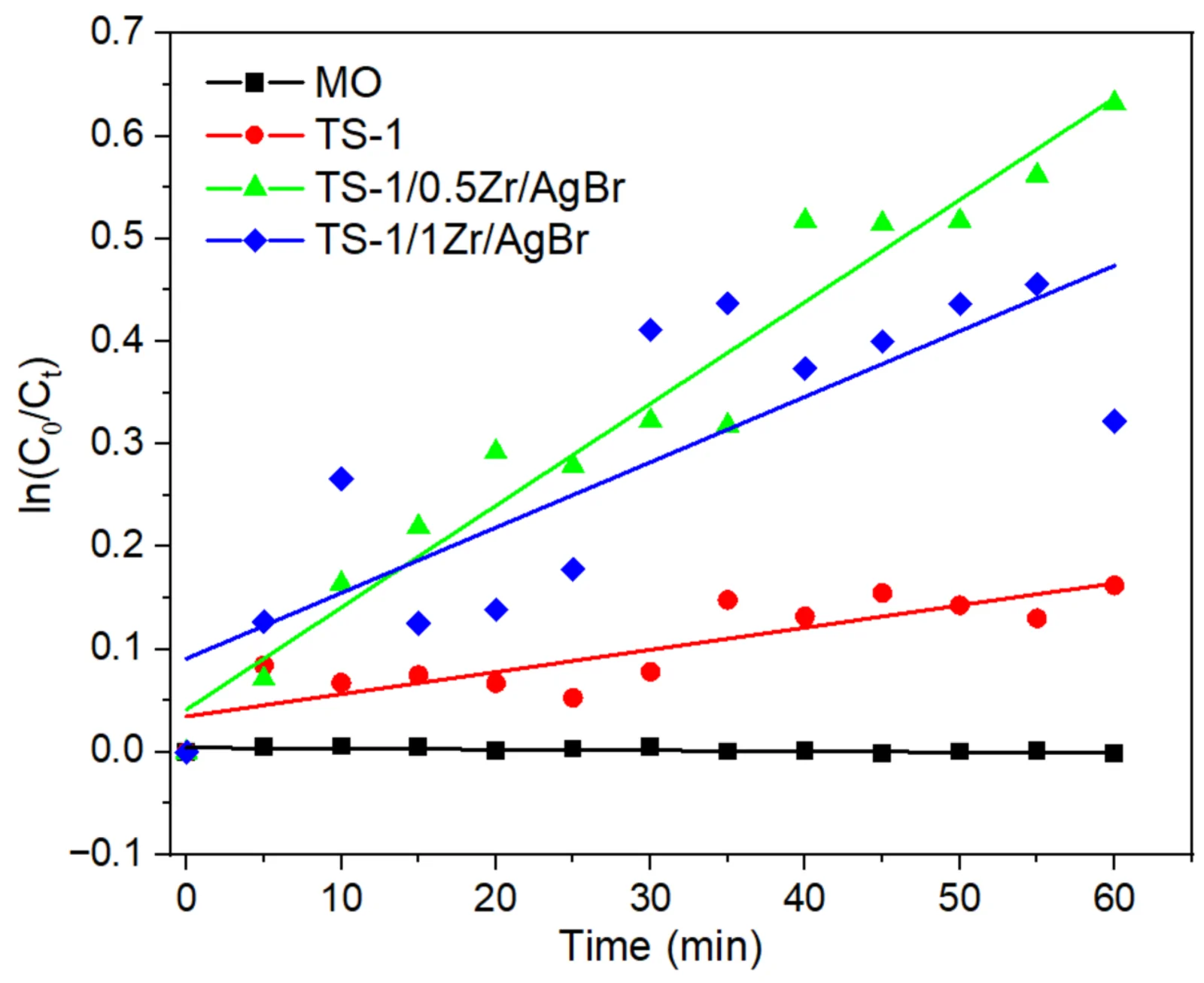
Pseudo-first-order kinetic plots of methyl orange (MO) removal under white-light irradiation in the absence of a catalyst (MO) and in the presence of TS-1, TS-1/0.5Zr/AgBr and TS-1/1Zr/AgBr. Symbols represent experimental data, and solid lines indicate linear regression fits of ln(C_0_/C_t_) versus irradiation time.

To evaluate the additional MO removal during the irradiation stage independently of the preceding dark adsorption, the MO concentration measured immediately before switching on the light was used as the initial concentration (C_0_) for the kinetic analysis. The corresponding ln(C_0_/C_t_) plots as a function of irradiation time are presented in Figure 9. The apparent pseudo-first-order rate constants (kapp) and the corresponding coefficients of determination (R^2^), obtained from linear regression of the kinetic data, are summarized in Table 3. Among the investigated materials, TS-1/0.5Zr/AgBr exhibited the highest apparent rate constant (0.009931 min^−1^, R^2^ = 0.9581), followed by TS-1/1Zr/AgBr (0.006385 min^−1^, R^2^ = 0.6666) and the parent TS-1 support (0.002162 min^−1^, R^2^ = 0.7421). The photolysis control showed a negligible apparent rate constant (−0.000087 min^−1^, R^2^ = 0.4241), indicating minimal changes in MO concentration in the absence of a catalyst. These results suggest that AgBr incorporation enhances MO removal during white-light irradiation compared with the unmodified TS-1 support.

**Figure 10:**
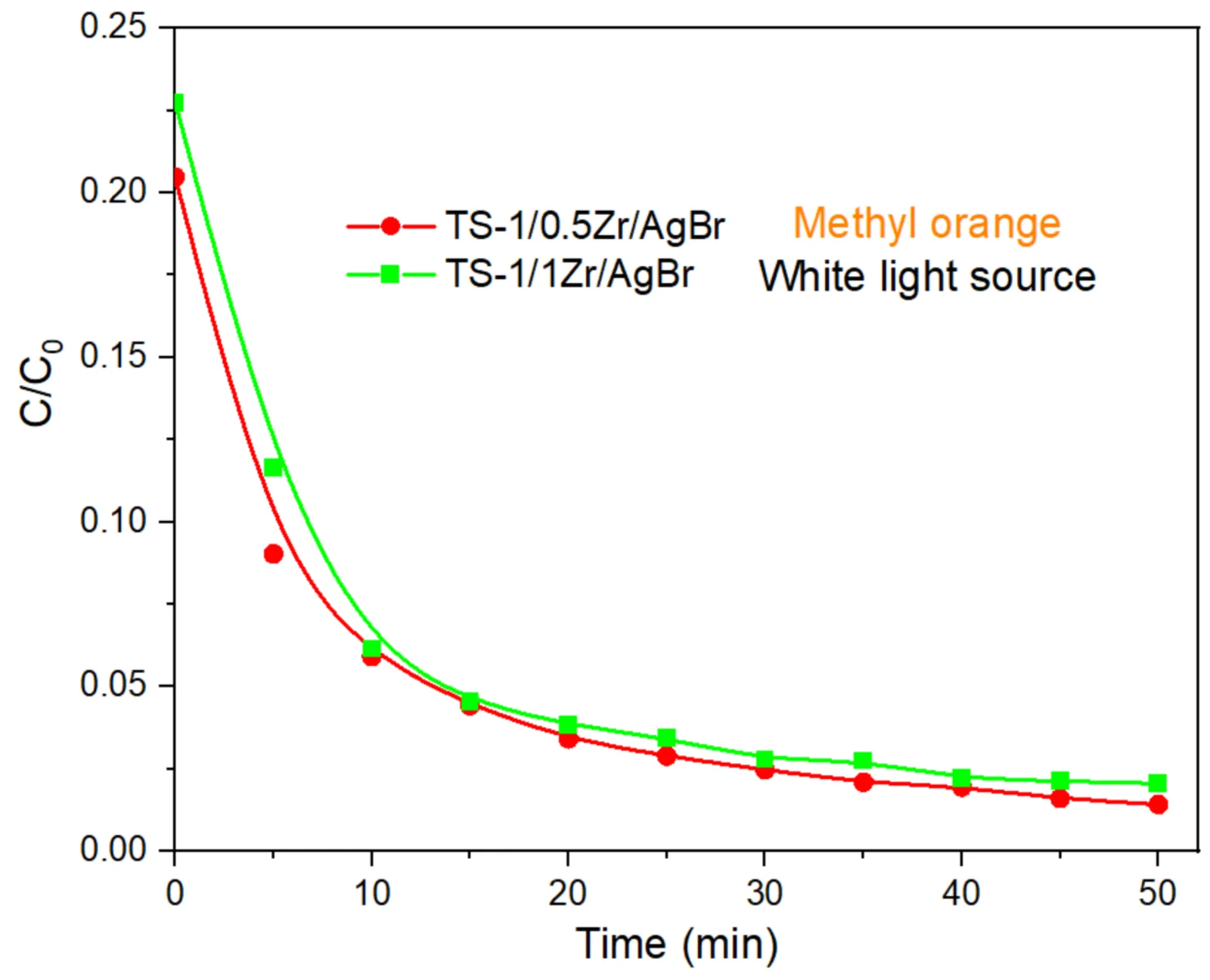
Photocatalytic degradation of methyl orange over TS-1/0.5Zr/AgBr and TS-1/1Zr/AgBr under white-light irradiation.

The photocatalytic performance of TS-1/0.5Zr/AgBr and TS-1/1Zr/AgBr was evaluated through the degradation of methyl orange (MO) under white-light irradiation, and the results are presented in Figure 10. As shown in Figure 10, both photocatalysts exhibited excellent activity toward MO degradation, reflected by the continuous decrease in the normalized concentration (*C/C_0_*) with increasing irradiation time. A rapid degradation stage was observed during the first 10 min of irradiation, indicating efficient generation of reactive species and effective interaction between the dye molecules and the photocatalyst surface. After 50 min of white-light irradiation, the normalized residual MO concentrations (*C/C_0_*, dimensionless) decreased to approximately 0.014 and 0.020 for TS-1/0.5Zr/AgBr and TS-1/1Zr/AgBr, respectively. These values correspond to MO removal efficiencies of approximately 98.6% and 97.9%, respectively. The slightly superior performance of TS-1/0.5Zr/AgBr can be attributed to its somewhat higher specific surface area and microporous accessibility, which provide a greater number of available active sites for photocatalytic reactions.

**Figure 11:**
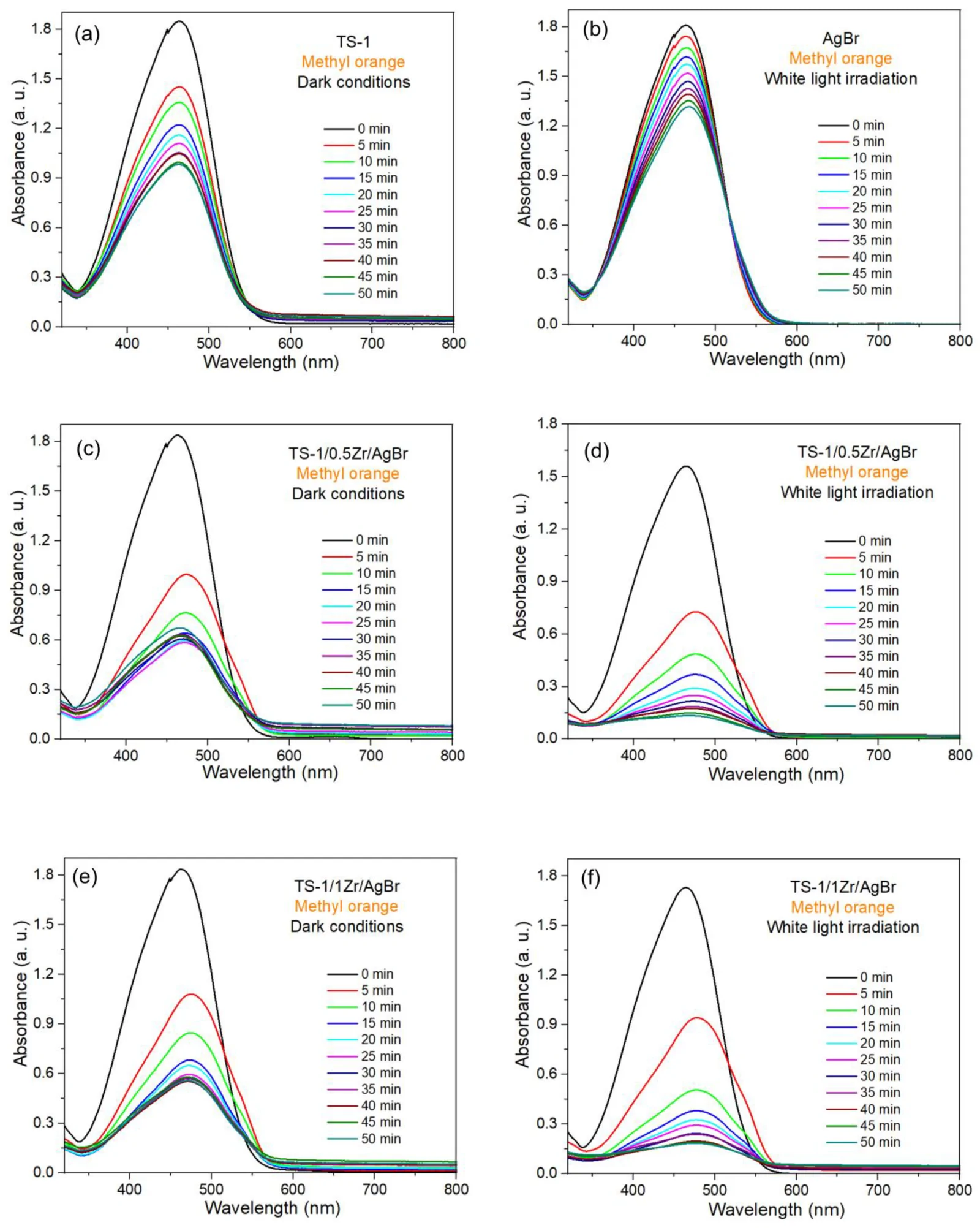
Time-dependent UV–Vis absorption spectra of methyl orange (MO) recorded in separate experiments under dark conditions in the presence of (**a**) TS-1, (**c**) TS-1/0.5Zr/AgBr, and (**e**) TS-1/1Zr/AgBr, and under white-light irradiation in the presence of (**b**) pure AgBr, (**d**) TS-1/0.5Zr/AgBr, and (**f**) TS-1/1Zr/AgBr. The dark adsorption and white-light irradiation experiments were conducted independently, without a preceding dark adsorption stage in the irradiation experiments.

The photocatalytic degradation process was further monitored by UV–Vis spectroscopy (Figure 11). For both photocatalysts, the characteristic absorption band of methyl orange located at approximately 465–470 nm gradually decreased with increasing irradiation time. The continuous reduction in absorbance confirms the progressive MO decolorization/degradation of MO chromophore under white-light irradiation. After 50 min, the absorption peak almost completely disappeared, indicating nearly complete dye removal from the solution. In contrast, pure AgBr exhibited a much slower decrease in absorbance, demonstrating inferior photocatalytic performance compared with the TS-1/Zr/AgBr composites. To assess the contribution of direct photolysis, a control experiment was performed using methyl orange (MO) under identical white-light irradiation conditions but without the addition of a photocatalyst. The UV–Vis absorption spectra recorded over 50 min showed no appreciable changes in the characteristic MO absorption band at approximately 464 nm (Figure S2), indicating that direct photolysis was negligible under the investigated conditions. Therefore, the pronounced decrease in MO absorbance observed in the presence of the AgBr-modified TS-1 composites cannot be attributed to direct photolysis alone. The Zr-free TS-1/AgBr composite exhibited substantial apparent MO removal (approximately 88%) (Figure S3), whereas removal efficiencies of approximately 98% were obtained for the investigated Zr-containing composites under the corresponding experimental conditions. These observations suggest that the Zr-containing formulations may exhibit improved overall MO removal performance. However, the measured Ag contents differ among the composites (10.91, 11.02, and 13.08 wt.%), and the observed removal includes contributions from adsorption and light-induced processes. Consequently, the present results do not permit the enhanced performance to be attributed exclusively to Zr incorporation. A more definitive assessment would require matched AgBr loadings and a systematic comparison of the adsorption and irradiation-stage contributions under identical experimental conditions.

The gradual decrease in the characteristic MO absorption band (–N=N–) demonstrates the effective destruction of the dye structure, which is generally attributed to cleavage of the azo bond during photocatalytic oxidation. Such behavior has been widely reported for methyl orange degradation in TiO_2_-based and related photocatalytic systems [33,34,35]. No significant shift in the absorption maximum was observed during the reaction, suggesting that the degradation proceeded without the accumulation of strongly absorbing intermediate products. These observations are in excellent agreement with the degradation profiles obtained from the *C/C_0_* measurements and further confirm the high photocatalytic efficiency of the AgBr modified TS-1/Zr composites. Overall, both materials demonstrated excellent photocatalytic performance under white-light irradiation, highlighting their potential for wastewater treatment applications. Notably, in contrast to our previous study [9], where the Zr-incorporated TS-1/0.5Zr and TS-1/1Zr samples exhibited negligible photocatalytic activity toward methyl orange degradation, the modified of AgBr resulted in a remarkable enhancement in photocatalytic performance, achieving degradation efficiencies above 98%. Dark-condition experiments (Figure 9) confirmed that adsorption also contributes to the removal of MO from the solution; however, the substantially higher removal observed under white-light irradiation demonstrates that adsorption alone cannot account for the high removal efficiencies. Thus, the enhanced performance of the Zr-modified TS-1/AgBr composites can be primarily attributed to their photocatalytic activity under visible-light irradiation, highlighting the crucial role of AgBr in promoting the photocatalytic degradation of MO.

**Figure 12:**
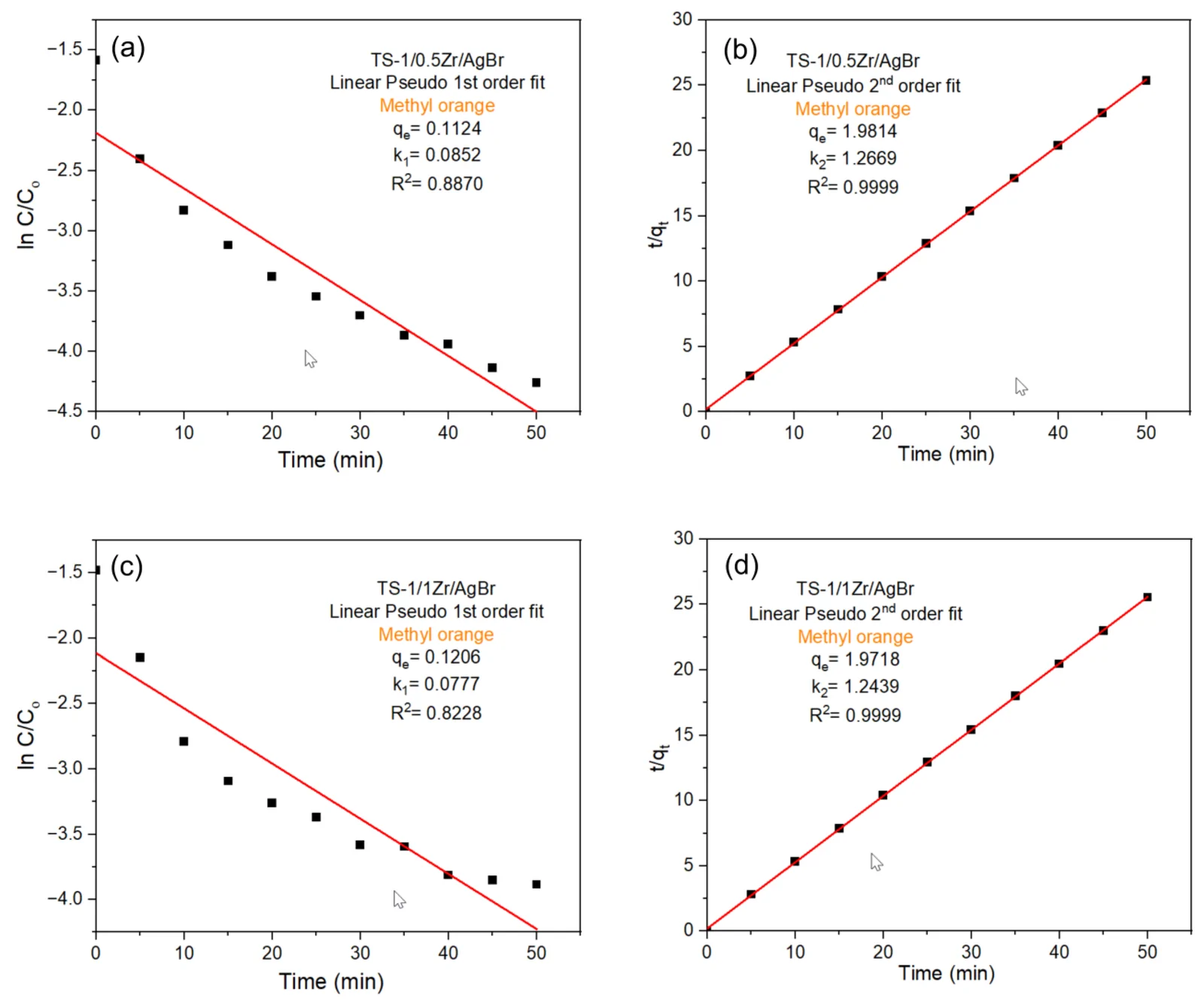
Kinetic analysis of methyl orange (MO) removal under white-light irradiation using pseudo-first-order and pseudo-second-order kinetic models: (**a**) pseudo-first-order plot of ln(C_t_/C_0_) versus irradiation time and (**b**) pseudo-second-order plot of t/q_t_ versus irradiation time for TS-1/0.5Zr/AgBr; (**c**) pseudo-first-order plot of ln(C_t_/C_0_) versus irradiation time and (**d**) pseudo-second-order plot of t/q_t_ versus irradiation time for TS-1/1Zr/AgBr. Symbols represent experimental data, and red lines indicate linear regression fits.

To further characterize the time-dependent removal of methyl orange (MO) under white-light irradiation, the experimental data were analyzed using pseudo-first-order and pseudo-second-order kinetic models. The corresponding linear plots and fitted parameters are presented in Figure 12. The pseudo-second-order model provided a better empirical fit to the experimental data, with correlation coefficients exceeding 0.9999 for both TS-1/0.5Zr/AgBr and TS-1/1Zr/AgBr. The fitted values of the apparent equilibrium removal capacities (q_e_) were 1.9814 and 1.9718 mg g^−1^, respectively, while the corresponding pseudo-second-order rate constants (k_2_) were 1.2669 and 1.2439. The similarity of these parameters indicates comparable time-dependent MO removal behavior under the investigated experimental conditions. However, since these experiments were conducted under irradiation without prior dark equilibration, the observed decrease in MO concentration may reflect contributions from both adsorption and light-induced processes. Therefore, the fitted kinetic parameters should be regarded as empirical descriptors of the overall MO removal rather than evidence of a specific adsorption or photocatalytic degradation mechanism.

This fitting behavior likely reflects the substantial contribution of adsorption and surface-mediated processes to the overall MO-removal behavior. However, the better fit obtained with the pseudo-second-order model should be regarded as an empirical description of the experimental data and should not be interpreted as evidence for a specific elementary photocatalytic reaction mechanism.

Overall, the kinetic analysis indicates that adsorption and surface-mediated processes contribute substantially to the observed MO-removal behavior. Compared with our previous study, in which TS-1/0.5Zr and TS-1/1Zr exhibited negligible photocatalytic activity toward methyl orange degradation [9], AgBr modification resulted in a marked enhancement of MO removal under white-light irradiation, demonstrating the important role of AgBr in the photocatalytic performance of the TS-1/Zr composites.

### 2.3. Catalyst Recycling

**Figure 13:**
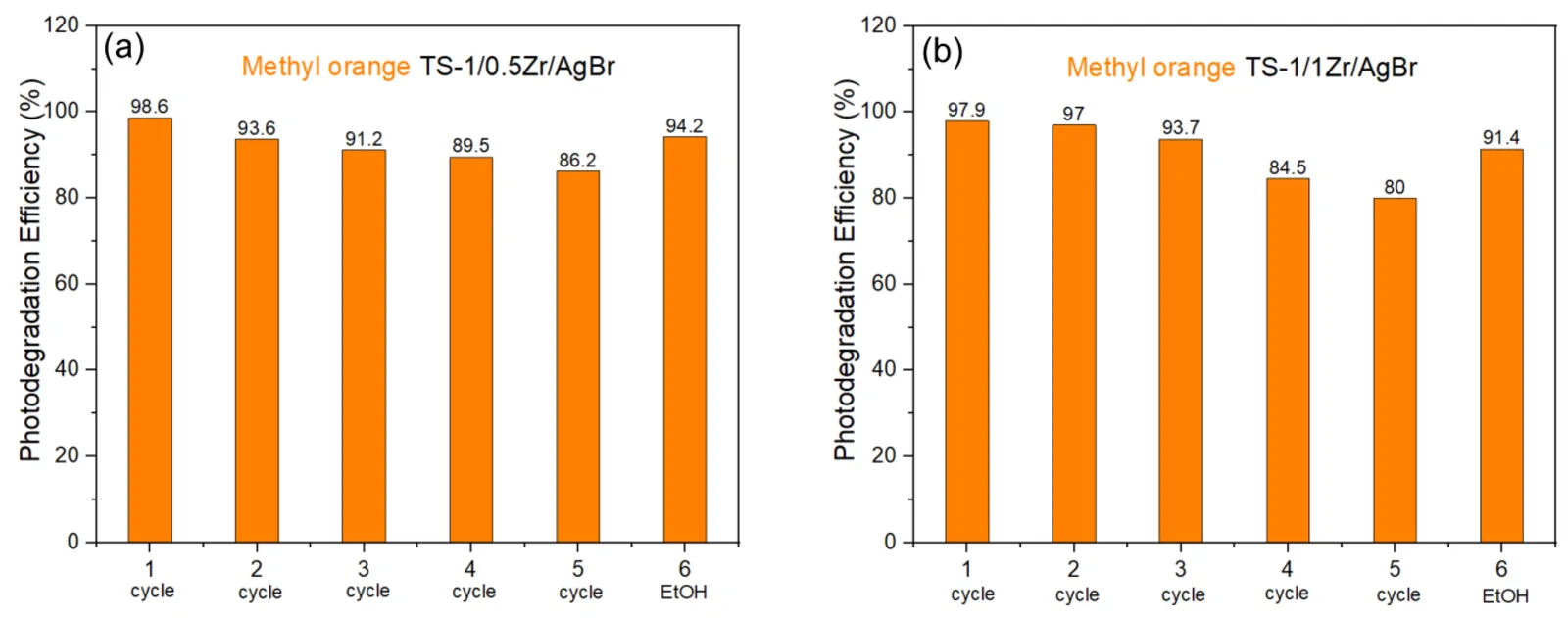
Reusability and stability of (**a**) TS-1/0.5Zr/AgBr and (**b**) TS-1/1Zr/AgBr photocatalysts during five consecutive photocatalytic degradation cycles of methyl orange under white-light irradiation. After the fifth cycle, the recovered catalysts were washed with ethanol (EtOH), resulting in a significant restoration of photocatalytic activity.

The reusability and stability of the synthesized TS-1/0.5Zr/AgBr and TS-1/1Zr/AgBr photocatalysts were evaluated through five consecutive photocatalytic degradation cycles of methyl orange under white-light irradiation (Figure 13). After each cycle, the photocatalyst was recovered by centrifugation, thoroughly washed with water, dried, and reused in the subsequent cycle. The masses of the recovered photocatalysts and their cumulative recovery yields are summarized in Table 4. For TS-1/0.5Zr/AgBr, the recovered catalyst mass decreased from an initial 50 mg to 18 mg after five successive recovery steps, corresponding to a cumulative recovery yield of 36%. Similarly, the recovered mass of TS-1/1Zr/AgBr decreased from 50 to 15 mg, corresponding to a cumulative recovery yield of 30%. These results indicate appreciable catalyst losses during repeated recovery and handling, which should be considered when interpreting the changes in photocatalytic performance.

The MO removal efficiency of TS-1/0.5Zr/AgBr decreased from 98.6% in the first cycle to 86.2% after the fifth cycle, while ethanol washing increased the observed removal efficiency to 94.2%. Similarly, TS-1/1Zr/AgBr exhibited a decrease from 97.9% to 80.0%, followed by an increase to 91.4% after ethanol treatment. The partial restoration of activity following ethanol washing is consistent with the possible removal of adsorbed species from the catalyst surface. However, the decrease in removal efficiency during successive cycles may reflect several contributing factors, including catalyst mass loss, surface fouling, and possible changes in the active AgBr component. The PXRD and FTIR results obtained after photocatalytic treatment (Figure 1b and Figure 2b) indicate that the principal structural features of the composites remain detectable after irradiation. Nevertheless, these measurements do not exclude minor surface modifications or AgBr photocorrosion. Overall, the recycling experiments demonstrate that both composites retain appreciable MO removal activity over repeated use, although the substantial loss of recoverable catalyst mass represents an important limitation for practical applications.

The recovery measurements reveal substantial cumulative catalyst losses during successive reuse cycles. Consequently, the observed decrease in MO removal efficiency may reflect both changes in the intrinsic properties of the recovered material and a reduction in the amount of catalyst available during subsequent experiments. The recyclability results should therefore be interpreted as the performance of the material under the applied recovery and reuse procedure, rather than as an isolated measure of intrinsic photocatalytic deactivation.

### 2.4. Plausible Photodegradation Mechanism of MO by TS-1/0.5Zr/AgBr and TS-1/1Zr/AgBr

**Figure 14:**
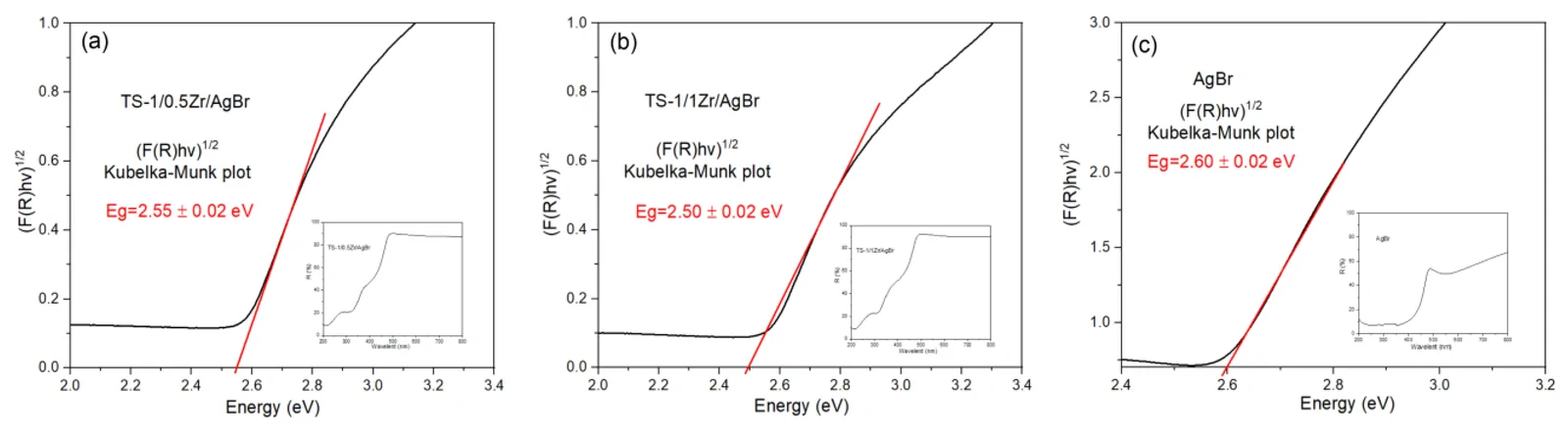
UV–Vis diffuse reflectance spectra of (**a**) TS-1/0.5Zr/AgBr, (**b**) TS-1/1Zr/AgBr and (**c**) pure AgBr. Insets show the Kubelka–Munk plots used for estimation of the apparent optical band-gap energies.

The optical properties of the AgBr-modified TS-1 catalysts were investigated by UV–Vis diffuse reflectance spectroscopy (Figure 14). Both samples exhibited strong absorption extending into the visible-light region owing to the modification of AgBr. The optical band-gap energies were estimated using the Kubelka–Munk transformation and the Tauc method by extrapolating the linear region of the (F(R)hν)^1/2^ versus photon energy plots [36]. The calculated band-gap energies were 2.55 ± 0.02 eV for TS-1/0.5Zr/AgBr and **2.50 ±** 0.02 eV for TS-1/1Zr/AgBr. The slightly lower band gap of TS-1/1Zr/AgBr indicates a marginal red shift of the absorption edge; however, the difference between the two samples is small, suggesting that the zirconium content has only a minor influence on the electronic structure of the AgBr-containing composites. For comparison, the band-gap energies determined for the pristine components are 2.60 ± 0.02 eV for AgBr (Figure 14) and TS-1 was reported in a previous article of ours [9]. The substantially wider band gap of TS-1 explains its limited response to visible light, whereas the incorporation of AgBr markedly shifts the optical response toward the visible region. The band-gap values of the composites, being close to that of AgBr, indicate that the optical absorption of the hybrid materials is predominantly influenced by the AgBr component. These relatively narrow band-gap energies are consistent with the excellent photocatalytic performance of both catalysts under white-light irradiation.

**Figure 15:**
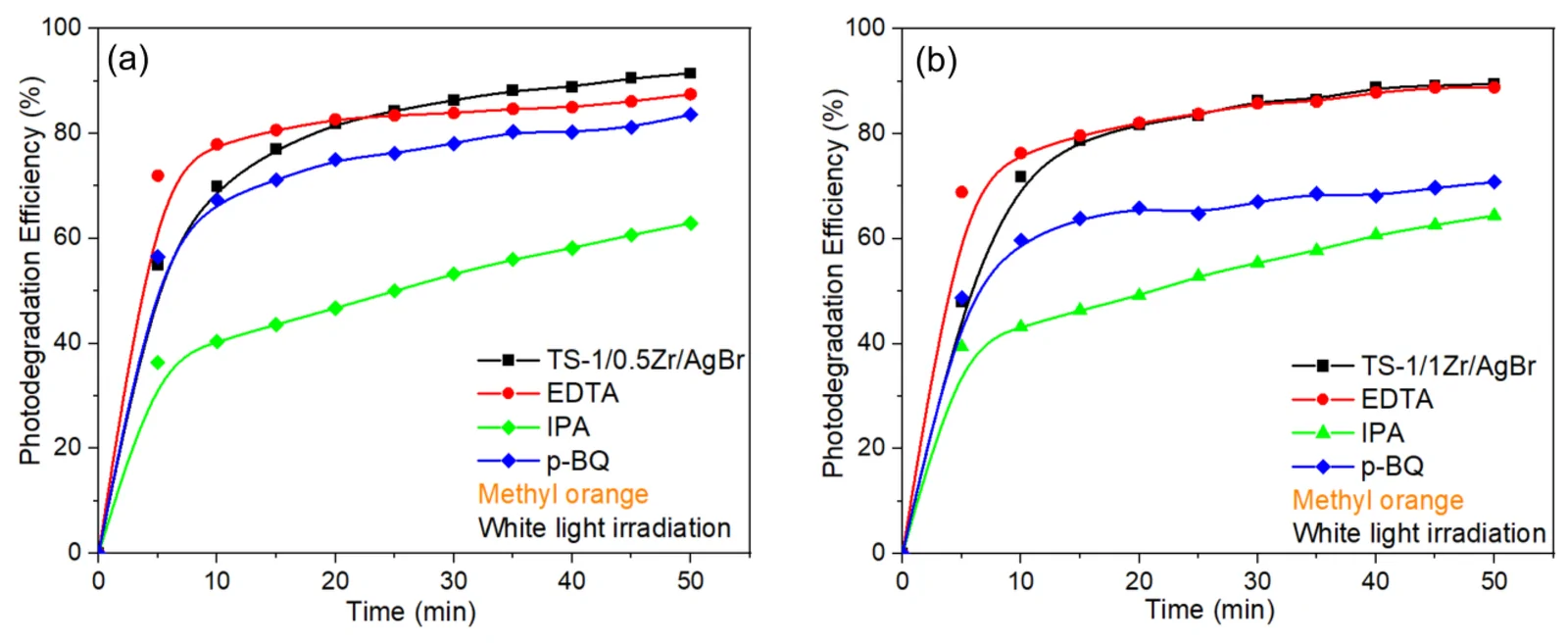
Effect of EDTA (10 mM), IPA (10 mM), and p-benzoquinone (p-BQ, 0.5 mM) on methyl orange removal under white-light irradiation in the presence of (**a**) TS-1/0.5Zr/AgBr and (**b**) TS-1/1Zr/AgBr. Irradiation was initiated immediately after the addition of the scavengers, without a preceding dark equilibration period. The observed removal efficiencies therefore reflect the combined contributions of adsorption and light-induced processes.

To investigate the possible involvement of reactive species in the photocatalytic removal of methyl orange (MO), scavenger experiments were performed using ethylenediaminetetraacetic acid (EDTA) as a hole (h^+^) scavenger, isopropanol (IPA) as a hydroxyl radical (•OH) scavenger, and *p*-benzoquinone (p-BQ) as a superoxide radical (O_2_•^−^) scavenger [37]. The scavengers, their stock concentrations, added volumes, and final concentrations in the reaction mixture are summarized in Table 5. The corresponding experimental results are presented in Figure 15. For both TS-1/0.5Zr/AgBr and TS-1/1Zr/AgBr, the addition of IPA produced the strongest inhibition of MO removal, decreasing the removal efficiency from approximately 90% in the absence of scavengers to 63–64% after 50 min of white-light irradiation. This pronounced inhibitory effect is consistent with an important contribution of hydroxyl radicals (•OH) to the observed photocatalytic process. The addition of p-BQ also reduced MO removal, with a more pronounced effect observed for TS-1/1Zr/AgBr, where the removal efficiency decreased from approximately 89% to 70%. This observation suggests that superoxide radicals (O_2_•^−^) may also participate in the reaction. In contrast, EDTA caused only a limited change in the removal efficiency of either composite, indicating that hole scavenging had a comparatively smaller influence under the investigated conditions. The similar response of both composites to IPA suggests that hydroxyl-radical-related pathways may be important in both systems, despite their different zirconium contents. Under white-light irradiation, excitation of the AgBr component may generate photogenerated charge carriers capable of participating in surface redox reactions and the formation of reactive oxygen species. These species may contribute to the disruption of the azo chromophore (–N=N–) of MO, consistent with the progressive decrease in its characteristic visible absorption band observed in the UV–Vis spectra. Overall, the scavenger experiments suggest that both •OH and O_2_•^−^ may contribute to MO removal, while the limited effect of EDTA indicates a less pronounced influence of hole scavenging under the investigated conditions. Nevertheless, scavenger experiments provide indirect mechanistic evidence, since the added compounds may also affect adsorption, surface reactions, and other processes in the reaction mixture. Therefore, the present results do not establish the relative contributions of individual reactive species quantitatively or provide direct evidence of a specific charge-separation pathway at the AgBr/TS-1 interface [35,38,39].

To evaluate the photocatalytic performance of the prepared composites, the degradation efficiency of TS-1/0.5Zr/AgBr and TS-1/1Zr/AgBr was compared with those of previously reported photocatalysts for methyl orange degradation under visible or white-light irradiation (Table 6). The TS-1/0.5Zr/AgBr composite achieved a degradation efficiency of 98.6%, while TS-1/1Zr/AgBr reached 97.9% after 50 min of irradiation. These values are comparable to or higher than those reported for most AgBr-based photocatalysts, including Ag/AgBr (72%), AgBr/g-C_3_N_4_ (87%), AgBr/WO_3_ (93.3%), AgBr–Ag_3_PO_4_ (80.7%), and Co_0.5_Zn_0.5_Fe_2_O_4_/AgBr (94.8%). Only a few photocatalysts, such as Bi_2_O_3_–NiO, Ag/AgBr/GO-U, 50% AgBr/BiOBr, and graphene/ZnO, have demonstrated complete (100%) degradation under their respective experimental conditions. Therefore, the present TS-1/Zr/AgBr composites exhibit competitive photocatalytic performance among recently reported AgBr-containing photocatalysts.

## 3. Materials and Methods

All reagents used for the synthesis of TS-1, TS-1/xZr, and TS-1/xZr/AgBr composites, as well as for the photocatalytic experiments, were purchased from Sigma-Aldrich (Schnelldorf, Germany) or Thermo Fisher Scientific (Waltham, MA, USA) and used without further purification. The reagents included tetraethyl orthosilicate (TEOS, 98%, Sigma-Aldrich), tetrapropylammonium hydroxide (TPAOH, 25 wt.% in water, Thermo Fisher Scientific), titanium (IV) isopropoxide (C_12_H_28_O_4_Ti, 98+%, Thermo Fisher Scientific), zirconium (IV) chloride (ZrCl_4_, 99.9%, Sigma-Aldrich), silver nitrate (AgNO_3_, ≥99%, Sigma-Aldrich), potassium bromide (KBr, ≥99%, Sigma-Aldrich), and methyl orange (MO, 85%, Sigma-Aldrich). Ultrapure water (electrical conductivity 0.055 μS cm^−1^, Q-FRONT EDI/BIO, Adrona, Riga, Latvia) was used throughout all synthesis and photocatalytic experiments.

### Synthesis of Zirconium-Substituted TS-1 (TS-1/xZr/AgBr)

**Scheme 1:**
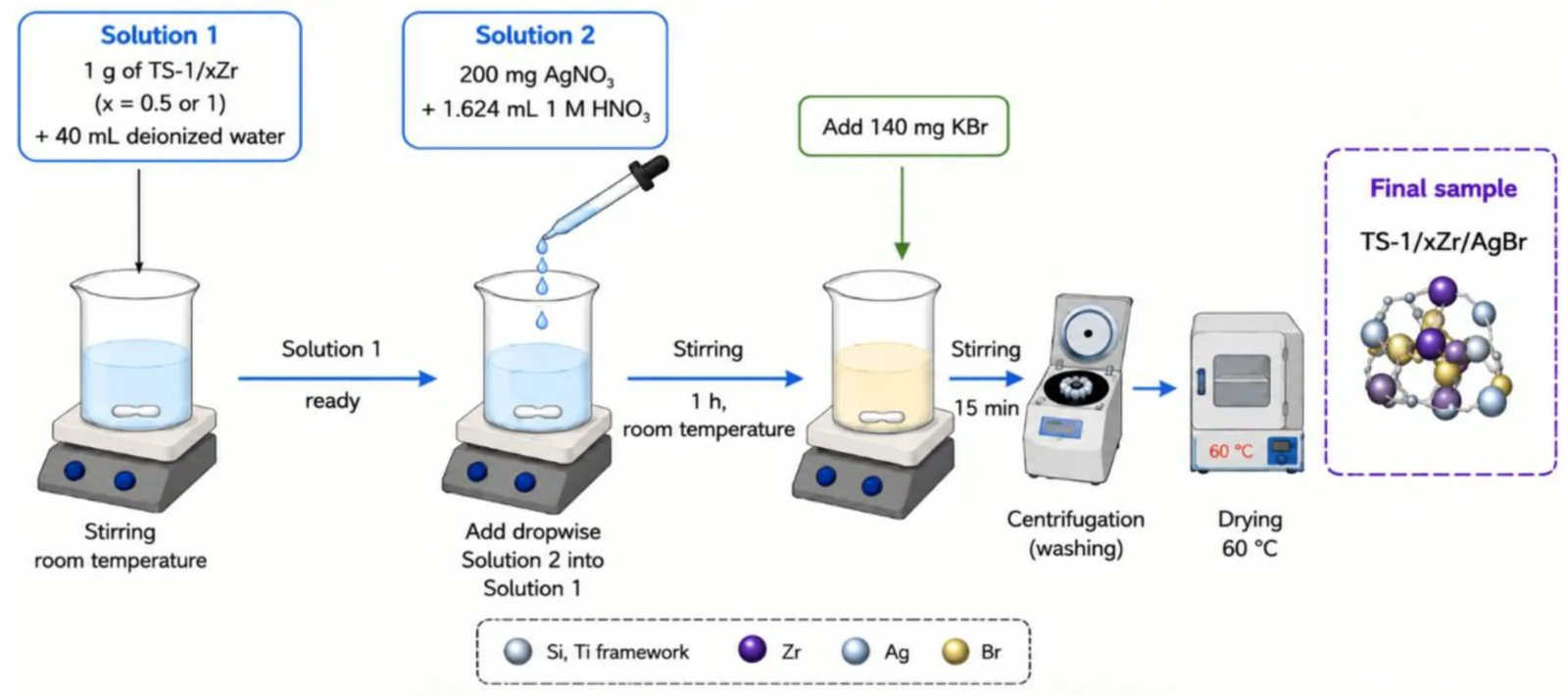
Preparation of AgBr-modified Zr-containing TS-1 composites.

The TS-1/0.5Zr and TS-1/1Zr materials employed as supports in the present investigation were prepared and characterized in our previous study [9]. The present work focuses on their subsequent modification with AgBr and the characterization and evaluation of the resulting TS-1/0.5Zr/AgBr and TS-1/1Zr/AgBr composites. Details of the synthesis and initial characterization of the Zr-containing TS-1 supports are available in Ref. [9]. To prepare the AgBr-modified samples, two Zr-incorporation materials (TS-1/0.5Zr and TS-1/1Zr) were used as starting solids. Solution 1 was prepared by dispersing 1.0 g of either TS-1/0.5Zr or TS-1/1Zr in 40 mL deionized water under stirring at room temperature. Solution 2 was obtained by dissolving 200 mg AgNO_3_ in 1.624 mL of 1 mol·L^−1^ HNO_3_. Solution 2 was added dropwise to Solution 1 under continuous stirring, after which the mixture was stirred for 1 h at room temperature. Subsequently, 140 mg KBr was added, and the suspension was stirred for an additional 15 min. The resulting solid was collected by centrifugation, washed, and dried at 60 °C (Scheme 1). The final materials were denoted as TS-1/0.5Zr/AgBr and TS-1/1Zr/AgBr, respectively.

Elemental composition was determined using an Epsilon-1 energy-dispersive X-ray fluorescence (EDXRF) spectrometer (Malvern Panalytical, Almelo, The Netherlands) equipped with Omnian software for standardless quantification (version 2.3.B/2022). Samples were finely ground and analyzed as loose powders. Measurements were performed under ambient conditions using an Ag-anode X-ray tube. The operating voltage and current were automatically optimized by the instrument software according to the elemental composition of the samples.

Powder X-ray diffraction (PXRD) measurements were carried out on an Empyrean diffractometer (Malvern Panalytical, Almelo, The Netherlands) equipped with a PIXcel3D detector and Cu Kα radiation (λ = 1.5406 Å). Diffraction patterns were recorded in the 3–90° 2θ range at 40 kV and 30 mA with a step size of 0.013°.

The textural properties of the materials were investigated using a 3Flex surface analyzer (Micromeritics, Norcross, GA, USA). Prior to analysis, the samples were degassed in situ at 90 °C for 5 h under vacuum (<1 × 10^−6^ mmHg). Nitrogen adsorption–desorption isotherms were measured at 77 K. The specific surface area (SBET) was calculated using the Brunauer–Emmett–Teller (BET) method in the relative pressure range of 0.05–0.31 P/P_0_ [61]. Total pore volume was estimated from the amount adsorbed at P/P_0_ = 0.96 [62]. Micropore volume was determined using the t-plot [63] and Horvath–Kawazoe methods [64], while pore-size distributions were calculated using the Barrett–Joyner–Halenda (BJH) model [65].

Fourier-transform infrared (FTIR) spectra were recorded using a Tensor 37 spectrometer (Bruker, Berlin, Germany). Samples were prepared as KBr pellets and analyzed in the 4000–400 cm^−1^ range with a resolution of 2 cm^−1^ and 128 accumulated scans.

Secondary electron (SE) images of the materials were carried out on ZEISS SEM EVO 25 LS—EDAX Trident (AMETEK GmbH, Weiterstadt, Germany) at acceleration voltage of 18 kV and beam current of 300–600 pA. The studied materials were fixed on pins using double-sided carbon tape and covered with carbon.

SEM-EDS mapping analyses were collected on a JEOL IT800SHL (Jeol Ltd., Tokyo, Japan). The two EDS detectors, (JED 2300, Jeol Ltd., Tokyo, Japan) 100 mm^2^ providing the possibility for high-sensitivity EDS analysis and acceleration voltage of 15 kV.

The TEM micrographs were registered by means of the Orius SC200D Model 833 CCD camera (Gatan Inc., Pleasanton, CA, USA) of the JEOL JEM 2100 HRTEM microscope (JEOL Ltd., Tokyo, Japan) at an accelerating voltage of 200 kV. In the preliminary preparation of TEM samples, the initial nanoparticle suspensions were dropped onto standard copper grids covered by amorphous carbon films and then dried under ambient conditions.

The photocatalytic activity of TS-1/0.5Zr/AgBr and TS-1/1Zr/AgBr was evaluated through the degradation of methyl orange (MO) under filtered white-light irradiation. The photocatalytic experiments were conducted using a 9 W white LED lamp with a color temperature of 4000 K, positioned approximately 5 cm above the reaction vessel. Continuous magnetic stirring was maintained throughout the irradiation period to ensure homogeneous dispersion of the photocatalyst particles in the methyl orange solution. In a typical experiment, 50 mg of photocatalyst was dispersed in 10 mL of a 20 mg L^−1^ aqueous MO solution. At predetermined time intervals (t = 1, 2, 3, 4, 5, 10, 15 min, etc.), a 2 mL aliquot was withdrawn from the reaction suspension and centrifuged at 14,000 rpm for 1 min to separate the photocatalyst. The supernatant was analyzed by UV–Vis spectroscopy at the characteristic absorption wavelength of MO (λ_max = 464 nm). Immediately after each measurement, both the analyzed supernatant and the separated photocatalyst pellet were returned to the reaction vessel, thereby maintaining an approximately constant reaction volume and catalyst concentration throughout the experiment. The suspension was then thoroughly mixed before continuing the irradiation. Prior to irradiation, the suspension was magnetically stirred to ensure homogeneous dispersion of the catalyst. The concentration changes during irradiation were monitored by UV–Vis spectroscopy at the characteristic absorption wavelength of MO (λmax = 464 nm) using a Cary 4000 spectrophotometer (Agilent Technologies, Santa Clara, CA, USA). The degradation efficiency was calculated according to (1) [66,67]:

(1) $$\mathrm{Degradation}\mathrm{Efficiency}(\%)\;=\;\frac{\left(C_{0}\;-\;C\right)}{C_{0}}$$

where *C*_0_ and *C* represent the initial concentration and the concentration at a given irradiation time, respectively.

The synthesis yield was not quantitatively determined, as the primary focus of this work was on framework modification and photocatalytic performance. All materials were synthesized under identical conditions and exhibited comparable solid recovery after hydrothermal crystallization and calcination, indicating good reproducibility of the preparation procedure.

Reactive species trapping experiments were conducted under white-light irradiation using ethylenediaminetetraacetic acid EDTA, isopropanol (IPA), and p-benzoquinone (p-BQ) as trapping agents for photogenerated holes (h^+^), hydroxyl radicals (•OH), and super-oxide radicals (O_2_•^−^), respectively. The concentrations and volumes of the scavenger solutions used in these experiments are provided in Table 5. The final concentrations of EDTA, IPA, and p-BQ in the reaction mixtures were 10 mM, 10 mM, and 0.5 mM, respectively. The total reaction volume was maintained at 10 mL in all experiments.

## 4. Conclusions

In this work, AgBr-modified Zr-containing TS-1 composites were prepared and evaluated for methyl orange removal under white-light irradiation. PXRD and FTIR analyses showed that the MFI framework was largely preserved after AgBr modification, while EDXRF confirmed the presence of Zr, Ag, and Br in the composite materials. SEM and TEM observations indicated a relatively homogeneous distribution of AgBr nanoparticles over the TS-1-based support. Diffuse-reflectance analysis yielded optical band-gap energies of approximately 2.55 and 2.50 eV for TS-1/0.5Zr/AgBr and TS-1/1Zr/AgBr, respectively, with the visible-light response predominantly governed by the AgBr component.

The composites exhibited high MO-removal efficiencies, reaching 98.6% and 97.9% after 50 min of white-light irradiation in the experiments conducted without prior dark equilibration. The additional sequential dark-adsorption and irradiation experiments demonstrated that adsorption contributes substantially to the overall MO removal. Analysis of the irradiation stage using an apparent pseudo-first-order model indicated the highest rate constant for TS-1/0.5Zr/AgBr, although the quality of the fits varied among the investigated materials. The kinetic models applied to the independent irradiation experiments should be regarded as empirical descriptions of MO removal rather than direct evidence of a specific photocatalytic reaction mechanism.

Radical-scavenger experiments indicated a major contribution from hydroxyl radicals, whereas photogenerated holes played a less pronounced role. Both materials retained substantial activity over five consecutive cycles, and the partial recovery of activity after ethanol washing suggests that surface accumulation of adsorbed intermediates contributes to catalyst deactivation.

Overall, AgBr modification converts weakly photoactive Zr-modified TS-1 into a strongly visible-light-responsive MO-removal material, while the MFI framework remains largely preserved. These findings demonstrate that AgBr-modified TS-1/Zr composites are promising and stable visible-light photocatalysts for the removal of organic pollutants from aqueous environments.

## Figures and Tables

**Table 1:** EDXRF—Elemental analysis of the synthesized TS-1/xZr/AgBr samples. The chemical composition of the parent TS-1 support was previously reported in Ref. [9].

Samples	Si wt%	Ti wt%	Zr wt%	Ag wt%	Br wt%	K wt%
TS-1	91.53	8.47	–	–	–	–
AgBr	–	–	–	52.91	47.09	–
TS-1/0.5Zr/AgBr	69.45	4.68	0.32	11.02	14.44	0.10
TS-1/1Zr/AgBr	67.96	4.12	0.67	13.08	14.05	0.12

**Table 2:** Textural properties of TS-1 and AgBr-modified Zr-incorporation TS-1 composites determined from N_2_ adsorption–desorption measurements.

Sample	S_BET_ (m^2^ g^−1^)	S_micro_ (m^2^ g^−1^)	V_micro_ (cm^3^ g^−1^)	V_total_ (cm^3^ g^−1^)	Average Pore Diameter nm
TS-1	414.52	270.82	0.13	0.29	2.8
TS-1/0.5Zr/AgBr	330.15	204.65	0.10	0.23	2.7
TS-1/1Zr/AgBr	324.33	199.26	0.10	0.22	2.7

**Table 3:** Apparent pseudo-first-order rate constants (k_app_) and corresponding determination coefficients (R^2^) for methyl orange removal under white-light irradiation in the presence of different photocatalysts and in the absence of a catalyst (photolysis control).

Photocatalyst	k_app_ (min^−1^)	R^2^
MO (photolysis control)	−0.000087	0.4241
TS-1	0.002162	0.7421
TS-1/0.5Zr/AgBr	0.009931	0.9581
TS-1/1Zr/AgBr	0.006385	0.6666

**Table 4:** Recovered catalyst mass and cumulative recovery yield of TS-1/0.5Zr/AgBr and TS-1/1Zr/AgBr during successive photocatalytic reuse cycles.

Cycle	TS-1/0.5Zr/AgBr (mg)	Recovery (%)	TS-1/1Zr/AgBr (mg)	Recovery (%)
1	50	100	50	100
2	39	78	41	82
3	32	64	31	62
4	28	56	25	50
5	22	44	22	44
6	18	36	15	30

**Table 5:** Scavengers and their concentrations used in the reactive species trapping experiments.

Scavenger	Target Reactive Species	Stock Concentration	Added Volume	Final Concentration
EDTA	Holes (h^+^)	0.1 M	1.0 mL	10 mM
IPA	Hydroxyl radicals (•OH)	0.1 M	1.0 mL	10 mM
p-BQ	Superoxide radicals (O_2_•^−^)	10 mM	0.5 mL	0.5 mM

**Table 6:** Comparison of the photocatalytic degradation efficiency of TS-1/Zr/AgBr composites with previously reported photocatalysts for methyl orange degradation.

Catalyst	Degradation Efficiency (%)	Ref.
Bi_2_O_3_-NiO	100.0	[40]
PANi-coated TiO_2_/SiO_2_	87.0	[41]
Ag/AgBr/GO-U	100.0	[4]
10% BaFe_12_O_19_/AgBr	98.2	[5]
Ag/AgBr	72.0	[12]
ZnFe_2_O_4_/AgBr/Ag	92.0	[13]
Ag/AgBr/RGO	88.0	[14]
AgBr/WO_3_	93.3	[16]
50% AgBr/BiOBr	100.0	[17]
Ag–AgBr/TiO2	92.0	[42]
In-AgBr	93.4	[43]
AgBr-MT-Br_2_	96.0	[44]
Ag/AgBr/(I/S)	92.0	[45]
AgBr/g-C_3_N_5_	63.1	[46]
AgBr/g-C_3_N_4_	87.0	[47]
graphene/ZnO	100.0	[48]
β-C_0_OOH/g-C_3_N_4_	92.1	[49]
WO_3_ nanosheets on g-C_3_N_4_ nanosheets	90.0	[50]
g-C_3_N_4_/Ag/γ-FeOOH	95.2	[51]
g-C_3_N_4_/Bi_2_WO_6_	94.8	[52]
BaTiO_3_@g-C_3_N_4_	76.0	[53]
Ag/AgBr/g-C_3_N_4_	90.0	[54]
Ag@AgBr/g-C_3_N_4_-70	95.0	[55]
AgBr/Ag_3_PO_4_	80.7	[56]
AgI/Ag/AgBr	97.0	[57]
AgBr/AgI@Ag	91.0	[58]
AgBr@Ag_3_PO_4_/Fe_3_O_4_	94.0	[59]
Co_0.5_Zn_0.5_Fe_2_O_4_/AgBr	94.8	[60]
TS-1/0.5Zr/AgBr	98.6	This work
TS-1/1Zr/AgBr	97.9	This work

## Data Availability

The original contributions presented in this study are included in the article. Further inquiries can be directed to the corresponding authors.

## Supplementary Materials

The following supporting information can be downloaded at: https://www.mdpi.com/article/10.3390/molecules31213921/s1, Table S1. UV–Vis absorbance of methyl orange at 464 nm during dark adsorption and subsequent irradiation; Table S2. Methyl orange removal during dark adsorption and subsequent visible-light irradiation; Figure S1. EDS spectra of sample TS-1/0.5Zr/AgBr; Figure S2. Time-dependent UV–Vis absorption spectra of methyl orange (MO) under white-light irradiation in the absence of a photocatalyst (photolysis control) over 50 min; Figure S3. Time-dependent UV–Vis absorption spectra of methyl orange (MO) in the presence of the Zr-free TS-1/AgBr composite under white-light irradiation. The progressive decrease in the characteristic MO absorption band indicates substantial removal of the dye from the aqueous phase during irradiation.

## Abbreviations

The following abbreviations are used in this manuscript:

MO	Methyl orange
PFO	Pseudo-first-order
PSO	Pseudo-second-order
