# Supplementary material for: Structural Characterization and Visible-Light Photocatalytic Performance of AgBr-Modified Zr-Containing Titanium Silicalite-1 (TS-1) Composites
Source: Molecules. 2026 Oct 30;31(21):3921. doi: 10.3390/molecules31213921 (PMC13764779; doi:10.3390/molecules31213921)
Supplement: Supplementary file 1 [file molecules-31-03921-s001.zip › molecules-4598303-supplementary.pdf]

Supplementary Materials

Structural Characterization and Visible-Light Photocatalytic Performance of AgBr-Modified Zr-Containing Titanium Silicalite-1 (TS-1) Composites

H. Lazarova[1,2,*], E. Tacheva[1,3], B. Barbov[1], R. Rusew[1,2], Stela Atanasova-Vladimirova[3,4], and B. Shivachev[1,*]

[1] Institute of Mineralogy and Crystallography "Acad. Ivan Kostov", Bulgarian Academy of Sciences, 1113 Sofia, Bulgaria: lazarova@imc.bas.bg (H.L.); tacheva_e@imc.bas.bg (E.T.); barbov@imc.bas.bg (B.B.); r.rusev93@imc.bas.bg (R.R.); bls@clmc.bas.bg (B.S.)

[2] PERIMED-2, BG16RFPR002-1.014-0007, Central District, Vasil Aprilov Blvd. 15A, 4002 Plovdiv, Bulgaria: lazarova@imc.bas.bg (H.L.); r.rusev93@imc.bas.bg (R.R.)

[3] National Centre of Excellence Mechatronics and Clean Technologies, 8 bul. Kliment Ohridski, P.C–1756 Sofia, Bulgaria: tacheva_e@imc.bas.bg (E.T.); (S.A.V.) statanasova@ipc.bas.bg

[4] Institute of Physical Chemistry, Bulgarian Academy of Sciences, 1113 Sofia, Bulgaria

* Correspondence: *lazarova@imc.bas.bg (H.L.); bls@clmc.bas.bg (B.S.)*

Table S1. UV–Vis absorbance of methyl orange at 464 nm during dark adsorption and subsequent irradiation 2

Table S2. Methyl orange removal during dark adsorption and subsequent visible-light irradiation 2

Figure S1. EDS spectra of sample TS-1/0.5Zr/AgBr. 2

Figure S2. Time-dependent UV–Vis absorption spectra of methyl orange (MO) under white-light irradiation in the absence of a photocatalyst (photolysis control) over 50 min. 3

Figure S3. Time-dependent UV–Vis absorption spectra of methyl orange (MO) in the presence of the Zr-free TS-1/AgBr composite under white-light irradiation. The progressive decrease in the characteristic MO absorption band indicates substantial removal of the dye from the aqueous phase during irradiation 4

Table S1. UV–Vis absorbance of methyl orange at 464 nm during dark adsorption and subsequent irradiation.

Sample	A_0	A_{50}	A_{55}	A_{60}	A_{120}
TS-1	1.8892	1.0535	1.0606	1.0419	0.8861
TS-1/0.5Zr/AgBr	1.9187	0.5886	0.5542	0.5481	0.2913
TS-1/1Zr/AgBr	1.8923	0.4920	0.4955	0.4973	0.3603

Note: A_0 represents the initial MO absorbance before catalyst addition. A_{50}, A_{55}, and A_{60} correspond to measurements during dark adsorption. A_{120} represents the absorbance after 60 min of irradiation following the 60-min dark adsorption period.

Table S2. Methyl orange removal during dark adsorption and subsequent visible-light irradiation

Sample	MO removal during dark adsorption (0–60 min), %	Additional MO removal during irradiation (60–120 min), %
TS-1	44.9	15.0
TS-1/0.5Zr/AgBr	71.4	46.9
TS-1/1Zr/AgBr	73.7	27.6

Note: Dark adsorption removal was calculated relative to the initial MO concentration before catalyst addition. Additional removal under irradiation was calculated relative to the MO concentration at the end of the 60-min dark adsorption period. The percentages are based on UV–Vis absorbance at 464 nm and represent apparent MO removal rather than confirmed mineralization.

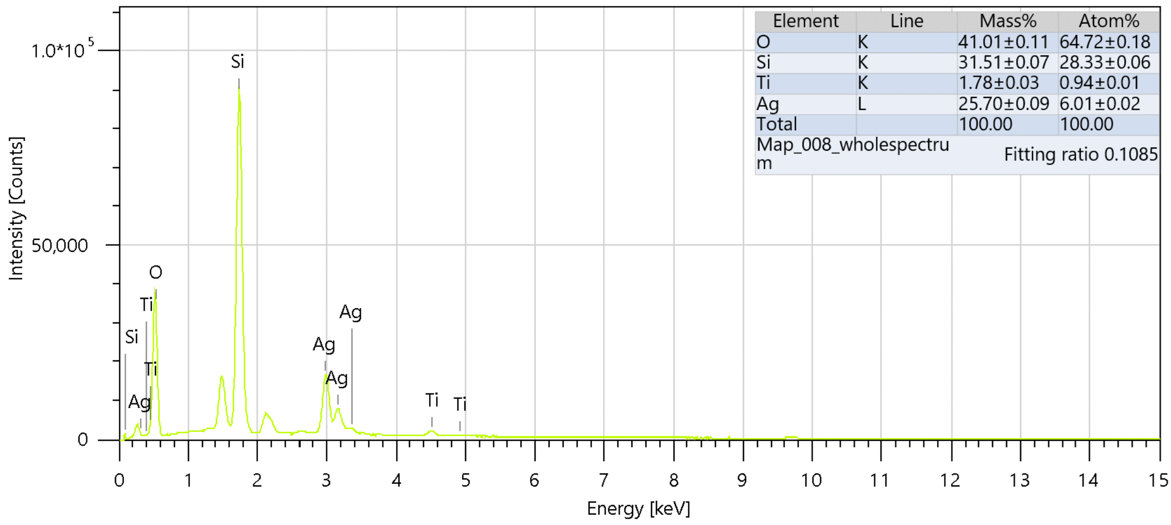

Figure S1. EDS spectra of sample TS-1/0.5Zr/AgBr.

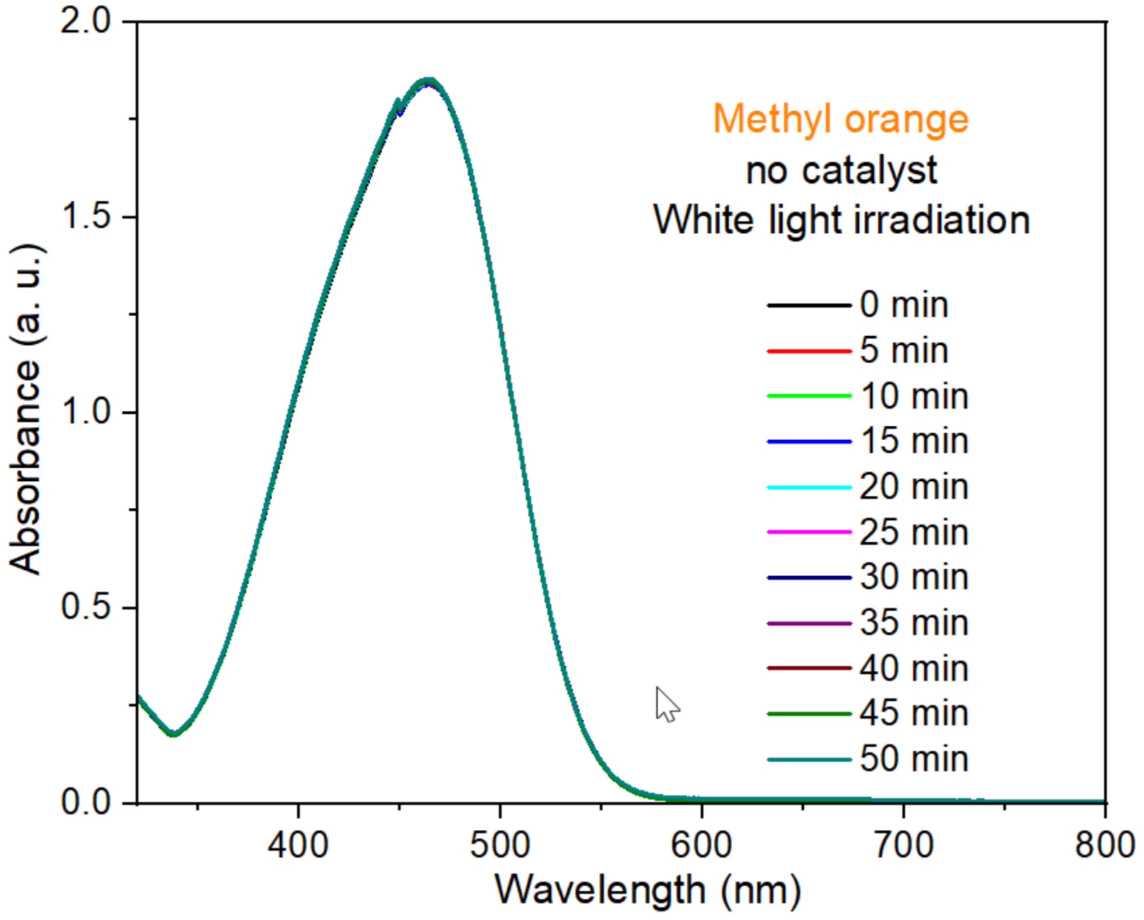

Figure S2. Time-dependent UV–Vis absorption spectra of methyl orange (MO) under white-light irradiation in the absence of a photocatalyst (photolysis control) over 50 min.

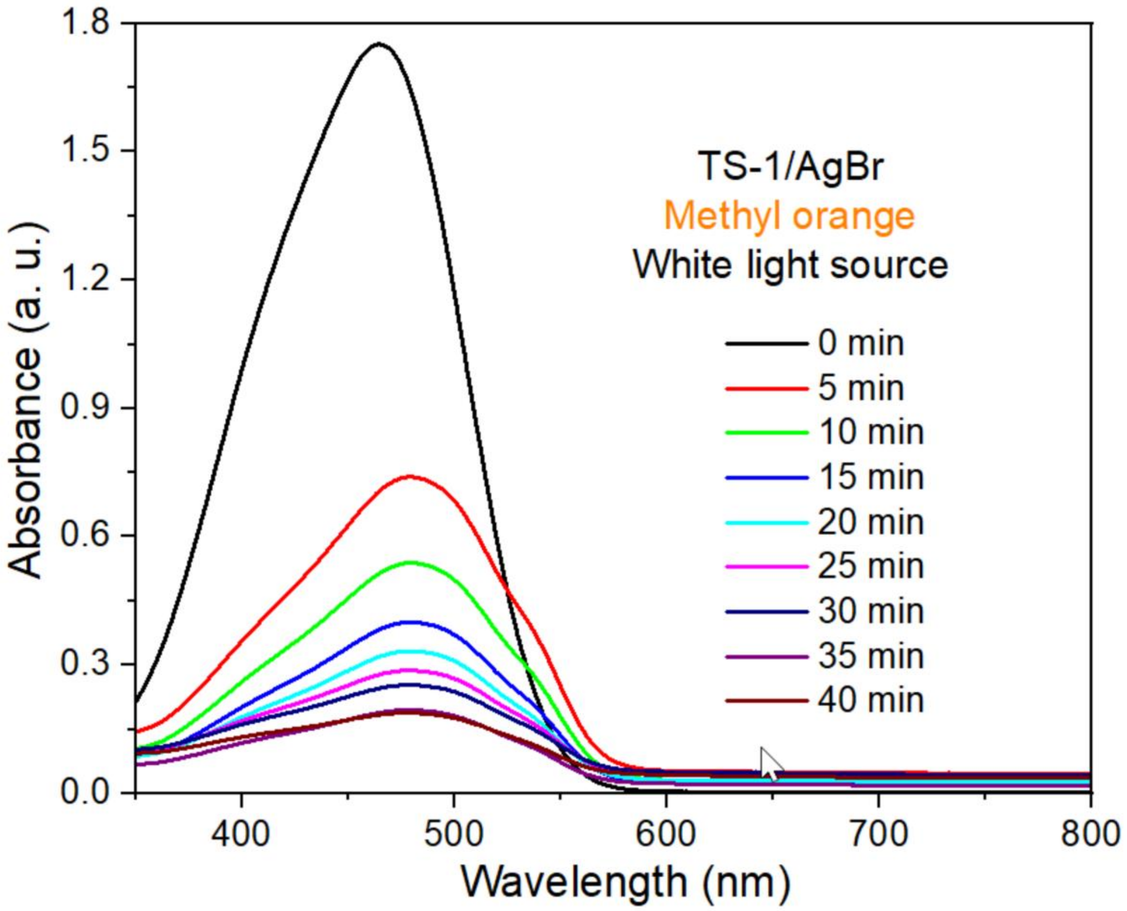

Figure S3. Time-dependent UV–Vis absorption spectra of methyl orange (MO) in the presence of the Zr-free TS-1/AgBr composite under white-light irradiation. The progressive decrease in the characteristic MO absorption band indicates substantial removal of the dye from the aqueous phase during irradiation.
